# PU.1-driven Th9 Cells Promote Colorectal Cancer in Experimental Colitis Models Through Il-6 Effects in Intestinal Epithelial Cells

**DOI:** 10.1093/ecco-jcc/jjac097

**Published:** 2022-07-06

**Authors:** Katharina Gerlach, Vanessa Popp, Stefan Wirtz, Ragheed Al-Saifi, Miguel Gonzalez Acera, Raja Atreya, Theresa Dregelies, Michael Vieth, Stefan Fichtner-Feigl, Andrew N J McKenzie, Frank Rosenbauer, Benno Weigmann, Markus F Neurath

**Affiliations:** Department of Medicine 1, Kussmaul Campus for Medical Research, University of Erlangen-Nuremberg, Erlangen, Germany; Department of Medicine 1, Kussmaul Campus for Medical Research, University of Erlangen-Nuremberg, Erlangen, Germany; Department of Medicine 1, Kussmaul Campus for Medical Research, University of Erlangen-Nuremberg, Erlangen, Germany; Medical Immunology Campus Erlangen, Friedrich-Alexander University Erlangen-Nuremberg, Erlangen, Germany; Department of Medicine 1, Kussmaul Campus for Medical Research, University of Erlangen-Nuremberg, Erlangen, Germany; Department of Medicine 1, Kussmaul Campus for Medical Research, University of Erlangen-Nuremberg, Erlangen, Germany; Department of Medicine 1, Kussmaul Campus for Medical Research, University of Erlangen-Nuremberg, Erlangen, Germany; Deutsches Zentrum Immuntherapie [DZI], Erlangen, University of Erlangen-Nuremberg, Germany; Institute of Pathology, Klinikum Bayreuth, Friedrich-Alexander University Erlangen-Nuremberg, Erlangen, Germany; Institute of Pathology, Klinikum Bayreuth, Friedrich-Alexander University Erlangen-Nuremberg, Erlangen, Germany; Department of General and Visceral Surgery, Medical Center-University of Freiburg, Freiburg, Germany; MRC Laboratory of Molecular Biology, Cambridge Biomedical Campus, Cambridge, UK; Laboratory of Molecular Stem Cell Biology, University of Münster, Münster, Germany; Department of Medicine 1, Kussmaul Campus for Medical Research, University of Erlangen-Nuremberg, Erlangen, Germany; Medical Immunology Campus Erlangen, Friedrich-Alexander University Erlangen-Nuremberg, Erlangen, Germany; Department of Medicine 1, Kussmaul Campus for Medical Research, University of Erlangen-Nuremberg, Erlangen, Germany; Deutsches Zentrum Immuntherapie [DZI], Erlangen, University of Erlangen-Nuremberg, Germany

**Keywords:** T cells, IL-9, colorectal cancer

## Abstract

**Background and Aims:**

Colorectal cancer [CRC] is one of the most frequent malignancies, but the molecular mechanisms driving cancer growth are incompletely understood. We characterised the roles of the cytokine IL-9 and Th9 cells in regulating CRC development.

**Methods:**

CRC patient samples and samples from AOM/DSS treated mice were analysed for expression of IL-9, CD3, and PU.1 by FACS analysis and immunohistochemistry. IL-9 citrine reporter mice, IL-9 knockout mice, and PU.1 and GATA3 CD4-Cre conditional knockout mice were studied in the AOM/DSS model. DNA minicircles or hyper-IL-6 were used for overexpression of cytokines *in vivo*. Effects of IL-6 and IL-9 were determined in organoid and T cell cultures. Claudin2/3 expression was studied by western blotting and bacterial translocation by FISH.

**Results:**

We uncovered a significant expansion of IL-9- and PU.1-expressing mucosal Th9 cells in CRC patients, with particularly high levels in patients with colitis-associated neoplasias. PU.1^+^ Th9 cells accumulated in experimental colorectal neoplasias. Deficiency of IL-9 or inactivation of PU.1 in T cells led to impaired tumour growth *in vivo*, suggesting a protumoral role of Th9 cells. In contrast, GATA3 inactivation did not affect Th9-mediated tumour growth. Mechanistically, IL-9 controls claudin2/3 expression and T cell-derived IL-6 production in colorectal tumours. IL-6 abrogated the anti-proliferative effects of IL-9 in epithelial organoids *in vivo*. IL-9-producing Th9 cells expand in CRC and control IL-6 production by T cells.

**Conclusions:**

IL-9 is a crucial regulator of tumour growth in colitis-associated neoplasias and emerges as potential target for therapy.

## 1. Introduction

The development of colorectal cancer [CRC], one of the most frequent malignancies worldwide, has been associated with chromosomal instability, microsatellite instability, and a CpG island methylator phenotype.^1,2^ Genetic and environmental factors contribute to the aetiology of familial and sporadic CRC.^1–3^. Additionally, the gut microbiota can induce colonic carcinogenesis through the induction of chronic intestinal inflammation.^4^ Furthermore, chronic inflammation in patients with inflammatory bowel diseases [IBD: Crohn´s disease, ulcerative colitis]^5–7^ may cause the development of colitis-associated CRC^8,9^ as an uncontrolled mucosal inflammation drives inflammation-associated neoplasia.^10–13^ Accordingly, risk factors for CRC development are the duration and the severity of inflammation in IBD. Suggestion was made that dysregulated intestinal homeostasis, characterised by mucosal damage and microbiota translocation followed by immune cell activation, is a key factor for the CRC development.^3,4^ Hereby, mucosal immune cells such as macrophages and lymphocytes produce pro-inflammatory, tumour-promoting cytokines like IL-6.^14^

IL-6 acts on epithelial cells and immune cells in CRC. It prevents apoptosis and favours cell proliferation. IL-6 binds to gp130 on target cells to activate the signal transducer and activator of transcription 3 [STAT3] in tumour cells and tumour-associated inflammatory cells ^12, 15^. During inflammation, IL-6 induces free radicals causing a DNA damage, favouring tumour initiation ^14^. Thus, IL-6 plays an important regulatory role for tumour initiation in CRC. However, the molecular mechanisms controlling IL-6 production remained poorly understood.

IL-9-producing CD4 T helper cells [Th9] were identified as a new subset of CD4 T helper cells with potent pro-inflammatory functions.^16–18^ In these cells, IL-9 gene expression is regulated by the cytokines TGFβ, IL-4, and IL-33 and transcription factors like PU.1, STAT6, GATA3, and IRF4.^19–22^ Th9 cells were identified in a variety of different chronic inflammatory and autoimmune diseases.^23–25^ In IBD, IL-9 was mainly produced by T cells in the lamina propria. In murine colitis models, IL-9 deficiency or PU.1 inactivation in T cells suppressed colitis activity. Anti-IL-9 antibodies also restrained colitis, suggesting that IL-9 could be used efficiently for colitis therapy ^24^.

Opposite to the function of IL-9 in chronic intestinal inflammation, little is known about the functional role of IL-9 in CRC. Unlike previous studies showing IL-9-dependent anti-tumour immune responses in melanoma,^26,27^ we detected that IL-9 plays a crucial role as a driver of tumour growth in an experimental model of CRC induced by azoxymethane [AOM]/dextran sodium sulphate [DSS] by regulating the IL-6 production by tumour-infiltrating T cells. These findings define a new role for IL-9 in CRC and highlight it as a potentially novel and highly interesting target for therapy.

## 2. Materials and Methods

### 2.1. Animals

Experiments with mice under specific pathogen-free [SPF] conditions were performed in accordance with institutional guidelines. IL-9-deficient Balb/c mice have been previously described. IL-9 reporter mice were generated by YouYi Hwang and described elsewhere.^24^ Mice with floxed PU.1 allele were generated by Frank Rosenbauer and were crossed to CD4-Cre lines. Conditional GATA3 mice were described elsewhere^28^ and crossed to the CD4-Cre lines.

### 2.2. Induction of colitis-associated neoplasias

Colitis-associated neoplasias were induced in IL-9 knockout mice, conditional PU.1 mice, conditional GATA3 mice, IL-9 citrine reporter mice, and wild-type [wt] controls by an intraperitoneal [i.p.] injection of AOM [10 mg per 1 kg body weight; Sigma] followed by three cycles of 2% DSS [MP Biomedicals] in drinking water for 1 week and normal drinking water for 2 weeks. Colitis and tumour development were monitored with the Coloview-system [Storz]. For the analysis of the IL-9 function, mice were injected with IL-9 minicircle vector DNA as described above. To investigate IL-6, 2 µg of hIL-6 were administered weekly intraperitoneally.

### 2.3. Induction model of intestinal inflammation

Colitis was were induced in WT mice by 2% dextran sodium sulphate [DSS; MP Biomedicals] in drinking water for 1 week. Wild-type mice were given an intraperitoneal injection of 80 μg anti-IL-9 antibodies [MM9C1; BioXCell] at various time points after the onset of colitis.

### 2.4. High-resolution mini-endoscopy and histopathology.

The development of colitis was monitored at various time points with a high-resolution video endoscopic system [Storz]. Murine Endoscopic Index of Colitis Severity [MEICS] scores for colitis severity were assigned at the end of the experiment on the basis of five parameters [translucency, granularity, fibrin, vascularity, and stool] according to an established scoring system ^29–31^.

### 2.5. Construction of IL-9 minicircles

An expression construct for *in vivo* overexpression of IL-9 was generated by cloning cDNA fragments encoding for murine full-length IL-9 into a minicircle. Minicircle DNA was produced using the MC-Easy DNA Production Kit [Systems Biosciences] and isolated with Qiagen Plasmid Maxikits including endotoxin removal. DNA was treated with the Miraclean Endotoxin Removal Kit [MirusBio]; 1–2.5 µg of minicircle DNA were administrated in Krebs–Ringer solution to mice via hydrodynamic injection.

### 2.6. Immunoflourescence staining of cryosections

Cryosections of colonic tissue were stained with H&E [haematoxylin [DAKO] and eosin [Sigma-Aldrich]]. For immunofluorescence staining, tissue sections were fixed in 4% paraformaldehyde/PBS or methanol, followed by Protein Block [DAKO]. For intracellular markers, sections were permeabilised with perm buffer [Ebioscience] for 20 min at 18°C. Staining was performed with the following primary antibodies at 4°C overnight: anti-CD3 [Ebioscience, 1:50], anti-IL-9R [Acris, 1:50], anti-Ki67 [DAKO, 1:50], anti-PU.1 [ThermoScientific, 1:50], anti-IL-9 [Biolegend, 1:50], anti-CD4 [Biolegend, 1:200], anti-IL-6 [Biolegend, 1:50], anti-claudin2 [1:200, Invitrogen], anti-claudin3 [1:100, Invitrogen], anti-SOCS3 [Invitrogen, 1:50], and anti-pSTAT3 [Cell Signaling, 1:50]. For staining of human cryosections the following antibodies were used: anti-CD3 [Ebioscience, 1.50], anti-IL-9 [BioLegend, 1:50], anti –PU.1 [ThermoScientific, 1:50], and anti-CD4 [Biolegend, 1:50]. Sections were then incubated with the secondary antibodies anti-rabbit ^Alexa488^, anti-rabbit ^Alexa594^, anti-rat ^Alexa488^, or anti-rat ^Alexa555^ [Invitrogen] and counterstained with DAPI [Vektor]. Image acquisition was performed on a confocal microscope [Leica]. Positive cells in high-power fields were counted in all samples per condition. Antibodies used are registered in Supplementary Figure 2].

### 2.7. Isolation of lamina propria mononuclear cells and epithelial cells

Lamina propria mononuclear cells [LPMCs] were isolated via LPMC dissociation-kit [Miltenyi] according to the manufacture´s guidelines. Epithelial cells were stimulated for 1 h with 1 µg recombinant IL-9. LPMCs were cultured at a density of 2.5 × 10^6^/ml in RPMI [10% FCS, 1% Pen/Strep, 1% L-Glutamin]. Cells were stimulated with anti-CD3 and anti-CD28 antibodies [4 µg/ml]. For *in vitro* analysis, cells were incubated for 24 h with 1 µg recombinant IL-9.

### 2.8. FACS analysis

Isolated cells were stained with rat-anti-mCD4^PECy7^, rat-anti-mCD19^APC^, rat-anti mCD8^PE^, rat-rat-anti-mIL-9R^PE^, rat-anti-mEpCAM^APC^, rat-anti-mEpCAM^PE^, rat-anti-mCD3^APC^, or mouse-anti-hCD3^APC^. Intracellular staining was done with rat-anti-mGATA3^APC^, rat-anti-mPU.1^APC^, rat-anti-mFoxP3^APC^, rat-anti-mRORγt^APC^, rat-anti-mTbet^APC^, rat-anti-mIL-6^PE^, rat-anti-mIL-9^PE^, rat-anti-mpSTAT3^APC^, rabbit-anti-mpSTAT1^APC^, anti-rabbit-mSOCS1^PE^, rabbit-anti-mSOCS3^PE^, or mouse-anti-hIL-9^PE^ together with permeabilisation buffer. Cells were analysed by FACS [BD Accuri] and percentage of positive cells was determined. Utilized antibodies are determined in [Supplementary Figure 3].

### 2.9. Western blot analysis of epithelial cells

Protein was extracted from murine tumour epithelial cells, and equal amounts were loaded onto 10% SDS page and transferred to a nitrocellulose membrane [BioRad]. After blocking for 1 h with TBS-T and 5% milk powder [Roth], detection of SOCS3 or claudin3 was acquired by using a specific antibody [Cell Signaling, 1:1000, or Invitrogen, 1:500] together with an anti-rabbit HRP antibody [Cell Signaling, 1:5000]. For detection of protein bands, the Pierce western blotting substrate ECL Plus [ThermoScientific] was used according to the manufacturer´s recommendations. Detection of ß-Actin [Santa Cruz, 1:5000] served as loading control.

### 2.10. Fluorescence *in Situ* hybridisation

The universal eubacterial oligonucleotide probe EUB-338 [GCT GCC TCC CGT AGG AGT] was labelled with Cy3. PFA-fixed cryosections of murine tumour tissue were incubated with 25 ng of each oligonucleotide added in 50 μl of hybridisation buffer containing 20% formamide for 90 min at 46°C before washing with the same stringency. Signal specificity was demonstrated by using *Escherichia coli* as positive control with the EUB-338 probe.

### 2.11. Isolation and imaging of intestinal crypts

For isolation of crypts, the fragments of the washed small intestine were incubated for 30 min at 4°C in PBS with 2 mM EDTA. The washed crypts were passed through a 70-µm cell strainer. Resuspended crypts with matrigel [50 µl] were applied in a pre-warmed 24-well plate. On the solified matrigel, 350 µl of IMDM medium were added. Incubation was done in a 37°C incubator with 5% CO_2_. The growth factors EGF, Noggin [R&D Systems], and R-spondin [PeproTech] and medium were exchanged every 3 days. After 5–7 days, crypts were passaged and 100 ng recombinant IL-9 [Immunotools], 100 ng hIL-6, or both were added until Day 10.

For immunofluorescent staining, matrigel was removed with Corning medium from washed and 4% PFA-fixed crypts. After blocking with protein block [DAKO], crypts were incubated with a specific monoclonal rat-anti-mKi-67 antibody [DAKO] or an anti-IL-9R antibody [Acris] overnight at 4°C, followed by staining with a secondary goat-anti-rat^Alexa555^ antibody [Life Technologies] [2h at room temperature]. Organoids were counterstained with DAPI [Vektor]. Organoids in mounting medium were applied on the glass bottom dish and analysed by confocal microscopy [Leica].

### 2.12. Isolation of colonic mRNA and real-time polymerase chain reaction analysis

Total RNA was isolated from colonic tissue with the RNA-Micro-kit [Machery-Nagel] according to the manufacture´s protocol. Total RNA [1 μg] was used to synthesise cDNA using iScript [Bio-Rad]. Quantitative real-time polymerase chain reaction **[**RT-PCR] was performed with SensiFAST SYBR [Bioline] and specific primers [Qiagen] on CFX-instruments [Bio-Rad]. Using 18S-rRNA, the relative expression level of cytokine mRNA was calculated with the formula: relative cytokine mRNA expression = 2^(ct[mRNA of interest] – ct[mRNA 18SrRNA]).

### 2.13. Patient material

The project was authorised by the local ethics committee of the University Erlangen-Nürnberg. Colonic specimens from patients with colorectal cancer, colitis associated cancer [CRC, CAC] were studied and compared with control samples. Control samples were taken from patients without any macroscopic or histological evidence of colitis or neoplasia. Patient characteristics, including patient numbers, age, and sex, are shown in Table 1.

**Table 1.** Patients’ characteristics.

Patient ID	Sex, age, number of cases	Age	Localisation	Disease
1	F	49	Colon sigma	C
2	F	43	Colon	C
3	M	55	Colon	C
4	F	29	Colon	C
5	M	25	Colon	C
6	M	26	Colon sigma	CRC
7	M	67	Colon sigma	C
8	M	78	Colon	C
9	F	73	Colon	CRC
10	F	73	ascendens	CRC
11	M	70	Colon sigma	CRC
12	F	74	Colon sigma	CRC
13	F	67	Colon sigma	CRC
14	M	70	Colon	CRC
15	M	80	C. descendens	CRC
16	F	82	Colon	CRC
17	F	41	Colon sigma	C
18	F	76	ascendens	CRC
19	F	35	Colon	CRC
20	F	35	Colon sigma	C
21	M	41	Colon	CAC
22	M	42	Colon	CAC
23	F	67	Colon	CAC
24	M	74	Colon	CAC
25	F	71	Colon	CAC
26	F	78	Colon	CAC
27	F	65	Colon	CAC
28	M	63	Colon	CAC
29	F	58	Colon	CAC
30	F	39	Colon	CAC
31	M	47	Colon	CAC
32	M	71	Colon	CAC
33	M	68	Colon	CAC
34	F	60	Colon	CAC
35	M	58	Colon	CAC
36	M	63	Colon	CAC
*n* = 36				
*n* C = 9				
*n* CRC = 11				
*n* CAC = 16				
Age [Ø] = 57				
Interval = [25–82]				
Female = 52, 70%				
Male = 47, 30%				

CAC, colitis-associated cancer; CRC, colorectal cancer; M, male; F, female.

### 2.14. Statistics

Statistical differences were determined by using Student’s t test; *p-*values <0.05 were considered as statistically significant and identified with asterisks. Results are expressed as mean values with error bars representing standard error of the mean [SEM]. Correlation studies were performed by Spearmen’s rho; *p*-values <0.05 were considered as statistically significant and identified with asterisks [*<0.05, **<0.01, ***<0.001]. The value for Spearmans rho is always between -1 and +1 and is interpreted as follows: >0 [monotone in the same direction]; ≈0 [no monotone connection]; <0 [opposite monotone relationship].

## 3. Results

### 3.1. Elevated IL-9 expression and increased numbers of Th9 cells in patients with colorectal cancer and colitis-associated cancer

**Figure 1.**
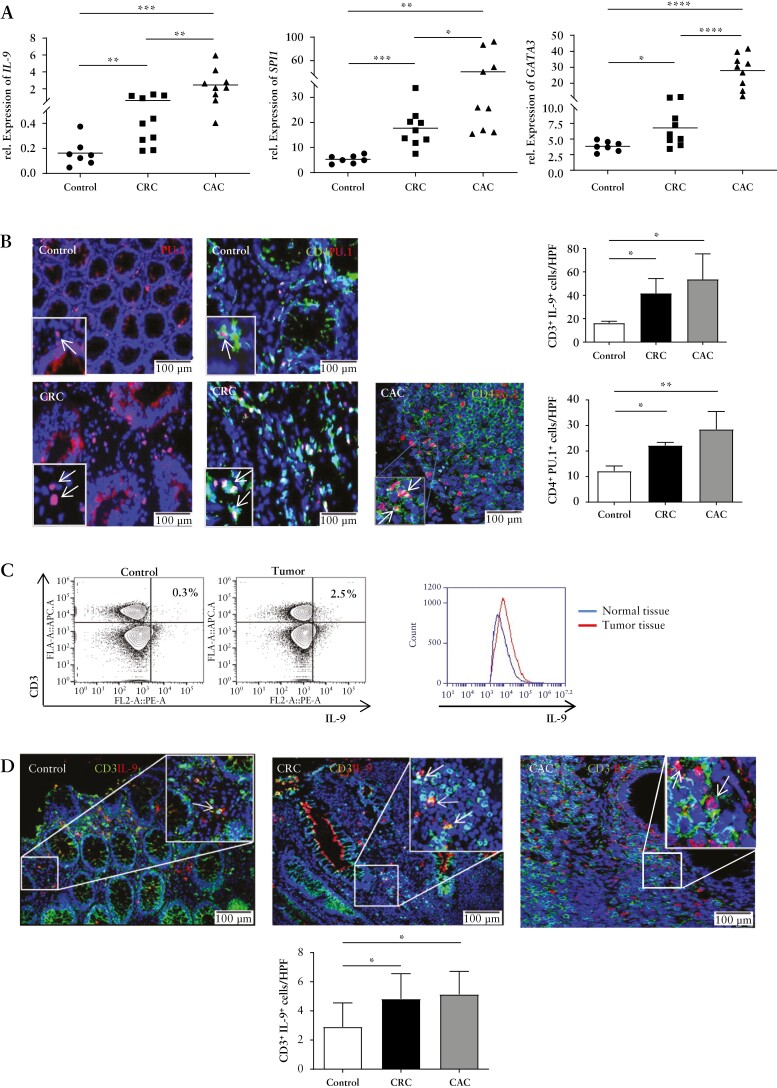
Elevated expression of PU.1 and IL-9 in patients with colorectal cancer. A] Total mRNA was isolated from patients with colorectal cancer, colitis-associated cancer [CRC, CAC] and healthy controls. Quantitative PCR analysis showed elevated mRNA levels of *SPI1*, *IL9*, and *GATA3* in tumour tissue. B] Immunofluorescence staining for PU.1 and CD4 from colorectal cancer patients and controls. Indicated cells were counted per high-power field [HPF] [lower panels]. Significant differences are indicated. Data represent mean values ± SEM per high-power field [*n* = 5–7]. C] FACS analysis of IL-9-expression in T cells in human colonic LPMCs from five tumour and control patients was performed. Representative scatter plots are shown. One representative overlay of IL-9 expression from LPMC T cells of colorectal cancer patients and controls is shown. Gating strategy is represented in the [Supplementary Fig 1]. D] Cryosections from colorectal cancer patients and controls were stained for CD3 and IL-9. Doublepositive cells per HPF and statistical analysis with significant differences [*p < 0.05; **p <0.01] of five patients is shown. CRC, colorectal cancer; CAC, colitis-associated cancer; SEM, standard error of the mean; LPMCs, lamina propria mononuclear cells.

To examine the potential relevance of IL-9 in patients with colorectal cancer, we addressed its expression in the presence or absence of tumour development. We noted significantly higher IL-9 mRNA levels in CRC and colitis-associated cancer [CAC] as compared with control patients [Figure 1A]. Elevated levels of the Th9-related transcription factors GATA3 and SPI1 were found in CRC and even more in CAC. Immunofluorescence double staining for CD4 and PU.1 revealed significantly more PU.1^+^ CD4^+^ T cells in the lamina propria of tumour patients [Figure 1B], suggesting that IL-9 producing cells in CRC and CAC are Th9 cells. Continuously, lamina propria cells showed more IL-9-expressing CD3^+^ T cells in the tumour tissue. Moreover, via immunofluorescent double staining, we detected more CD3 and IL-9 expressing cells in CRC and CAC [Figure 1C, D]. These findings clearly indicated the presence of Th9 cells in human colorectal cancer high levels were noted particularly in colitis-associated neoplasias.

### 3.2. Mucosal PU.1^+ ^T cells producing IL-9 are induced in experimental colorectal cancer

**Figure 2.**
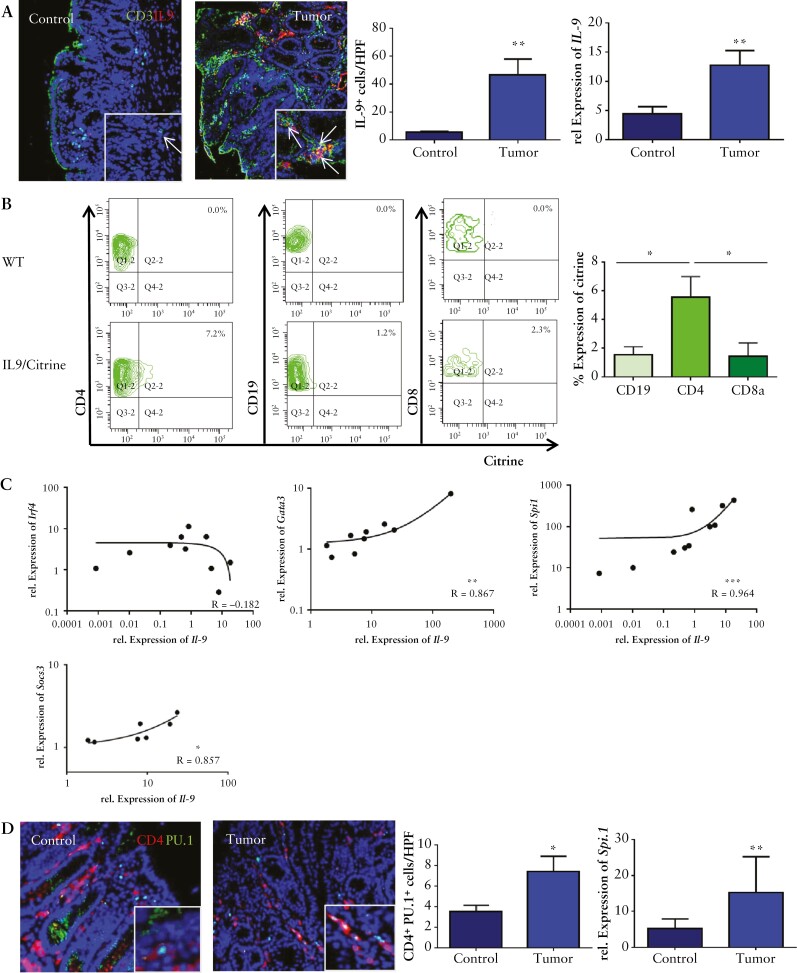
Th9 cells producing IL-9 are induced in CRC. A] Left panels: immunofluorescence staining for CD3 [green] and IL-9 [red] in the lamina propria of tumour tissue with representative staining, and statistical analysis is shown [*n* = 3]. Total mRNA from tumour tissue of AOM/DSS-treated wild-type mice and controls was isolated. Right panel: qPCR analysis showed a significant upregulation for *IL-9* mRNA levels in experimental CRC [*n* = 7]. Significant differences are indicated [**p <0.01]. B] Isolated LPMCs from tumour tissue of IL9 citrine reporter mice were stained for CD4, CD19, and CD8. Representative scatter plots are shown from one representative experiment out of two. Statistical analysis with significant differences [*n* = 3] is shown below [*p <0.05]. C] Relative mRNA expression levels for *Il-9* and *Gata-3*, *Spi1*, *Irf4*, and *Socs3* were analysed in CRC tissue from wild-type mice. Correlation coefficients are indicated. D] Left panels: immunofluorescence staining for CD4 [red] and PU.1 [green]. Double-positive cells were counted per HPF from six mice [right upper panel]. Right panel: qPCR analysis for the expression levels of *Spi1* was performed on tumour tissue and control tissue [*n* = 10] from wild-type mice. CRC, colorectal cancer, LPMCs, lamina propria mononuclear cells; HPF, high-power field.

To determine the potential presence of Th9 cells in experimental colorectal we investigated the expression of IL-9 in the AOM/DSS model.^32^ Quantitative PCR analysis revealed a significant induction of *IL-9* mRNA expression in isolated colorectal tumors from AOM/DSS treated wildtype [WT] mice as compared with untreated controls [Figure 2A, upper right panel]. Consistently, immunofluorescence analysis demonstrated a significant increase of mucosal IL-9-expressing T cells in colonic tumours, indicating the presence of IL-9 and Th9 cells [Figure 2A, left panel].

To analyse the cell type producing IL-9 in colorectal tumours more closely, we took IL-9 citrine reporter mice. Isolated LPMCs from AOM/DSS tumours were examined in terms of the expression of IL-9. We found that mainly CD4^+ ^T cells, rather than CD19^+ ^B cells and CD8^+ ^T cells, produce IL-9 in colorectal tumours [Figure 2B]. *IL-9* mRNA levels in tumours showed a marked correlation with the Th9-associated transcription factors *Irf4, Gata3*, and *Spi1*, as well as with *Socs3* [Figure 2C]. Consistently, numbers of CD4^+ ^PU.1^+ ^T cells were significantly increased in colorectal tumours, thereby suggesting the presence of Th9 cells [Figure 2D].

**Figure 3.**
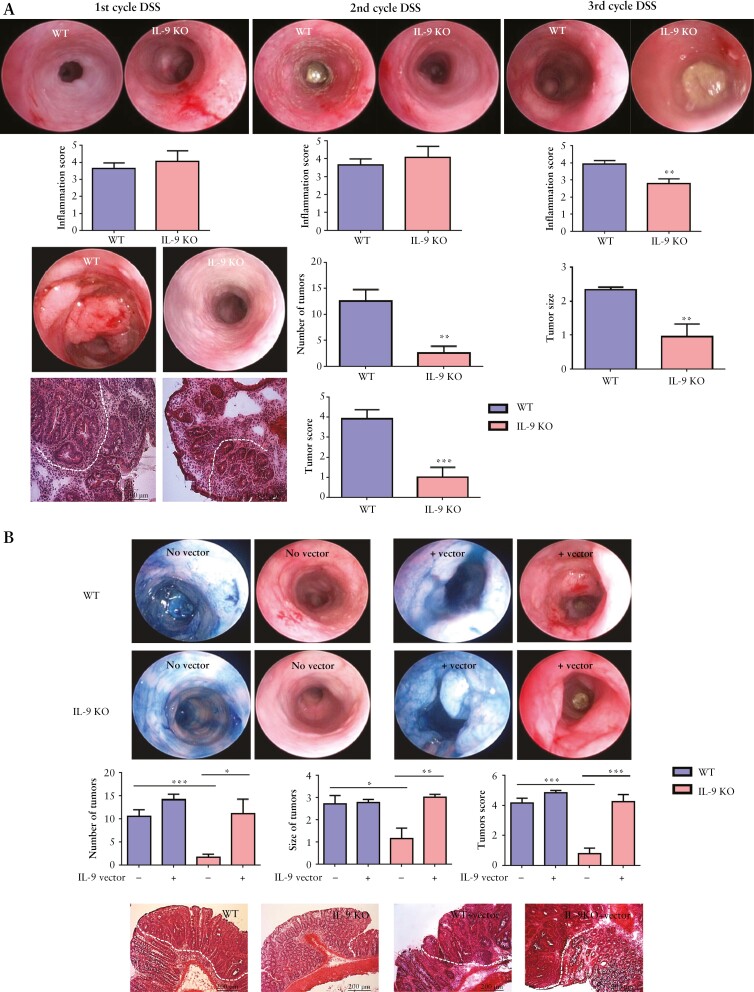

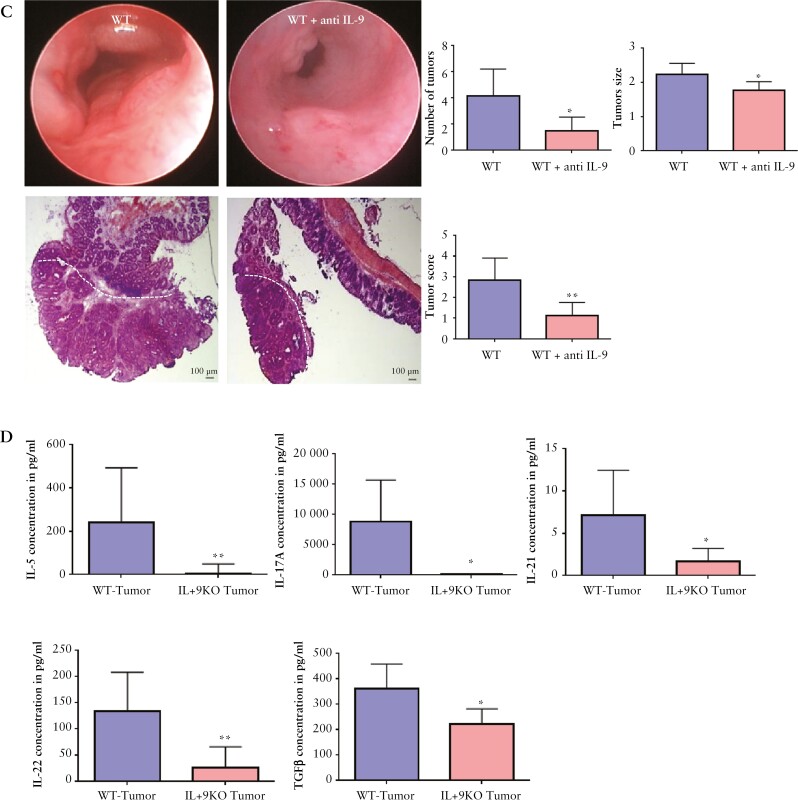
Central regulatory role of IL-9 and PU.1 in T cells in a murine CRC model. A] IL-9-deficient and wild-type mice in the AOM/DSS model. Inflammation was analysed after each DSS cycle. Representative pictures of the endoscopy and histopathology are shown. Data from three independent experiments [*n* = 18] with significant differences are indicated [**p <0.01; ***p <0.001]. B] AOM/DSS-treated wild-type [*n* = 15] and IL-9 knockout [*n* = 16] mice were treated by hydrodynamic injection with an IL-9 vector. Chromoendoscopy with methylene blue is shown. Significant differences are indicated [*p <0.05; **p <0.01; ***p <0.001]. C] Anti-IL-9 antibody therapy in WT mice treated with AOM/DSS. Mice were injected with an anti-IL-9 antibody before the induction of CRC. H&E stainings and statistical analysis with significant differences [*n* = 3] are shown [*p <0.05; **p <0.01]. D] Supernatants of tumour LPMCs from WT and IL-9-deficient mice were analysed for IL-5, IL-17A, IL-21, IL-22, and TGFβ production. Data represent results of 10 mice per group [*p <0.05; **p <0.01]. CRC, colorectal cancer; AOM, azoxymethane; DSS, dextran sodium sulphate; LPMCs, lamina propria mononuclear cells; WT, wild-type; H&E, haematoxylin and eosin.

To study the functional role of IL-9 in colorectal neoplasias, we used the AOM/DSS model in wild-type and IL-9 knockout mice. We observed equal signs of inflammation after the first cycle of DSS in both species. However, after the third cycle of DSS IL-9, deficiency led to a significantly decreased inflammation score. Tumour numbers, tumour size, and tumour score in IL-9 knockout mice were significantly reduced [Figure 3A]. Then, we explored the effect of IL-9 on tumour growth by expression via mini-circle DNA ^33^. Whereas the overexpression of IL-9 had no significant effect on the tumour growth in IL-9-proficient wild-type mice, IL-9 expression led to a significantly augmented tumour growth in IL-9-deficient animals [Figure 3B].

We next analysed the effect of an anti-IL-9 antibody on the development of colorectal cancer. Therefore, we treated wild-type mice with a blocking anti-IL-9 antibody followed by an AOM/DSS treatment. Mice receiving the anti-IL-9 antibody had fewer tumours and a significantly reduced tumour score as compared with controls [Figure 3C]. These findings further support the pro-tumour activity of IL-9.

Finally, we investigated the distribution of immune cells and the production of pro-inflammatory cytokines. We found no significant differences in CD4^+ ^and CD8^+ ^T cell numbers between IL-9 knockout mice and wild-type controls [data not shown], thus suggesting that IL-9 has no major effect on the presence of these T cell subsets in colorectal neoplasia. In contrast, IL-5, IL-17A, IL-21, IL-22, and TGFβ concentrations in the tumour tissue of IL-9 knockout mice were significantly reduced compared with wild-type mice [Figure 3D], indicating that IL-9 controls the production of Th2- and Th17-associated cytokines.

### 3.3. PU.1^+ ^T cells rather than GATA3^+ ^T cells are crucial inducers of colitis-associated cancer

**Figure 4.**
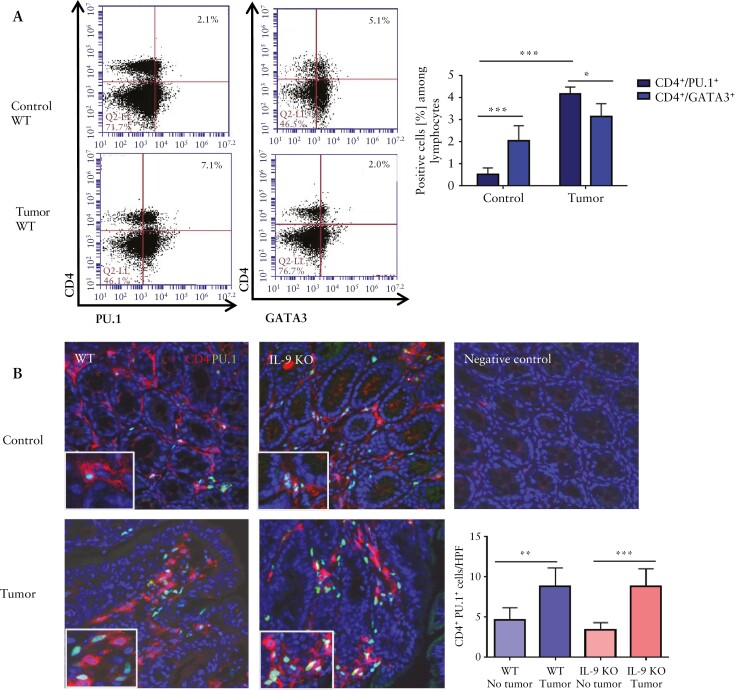

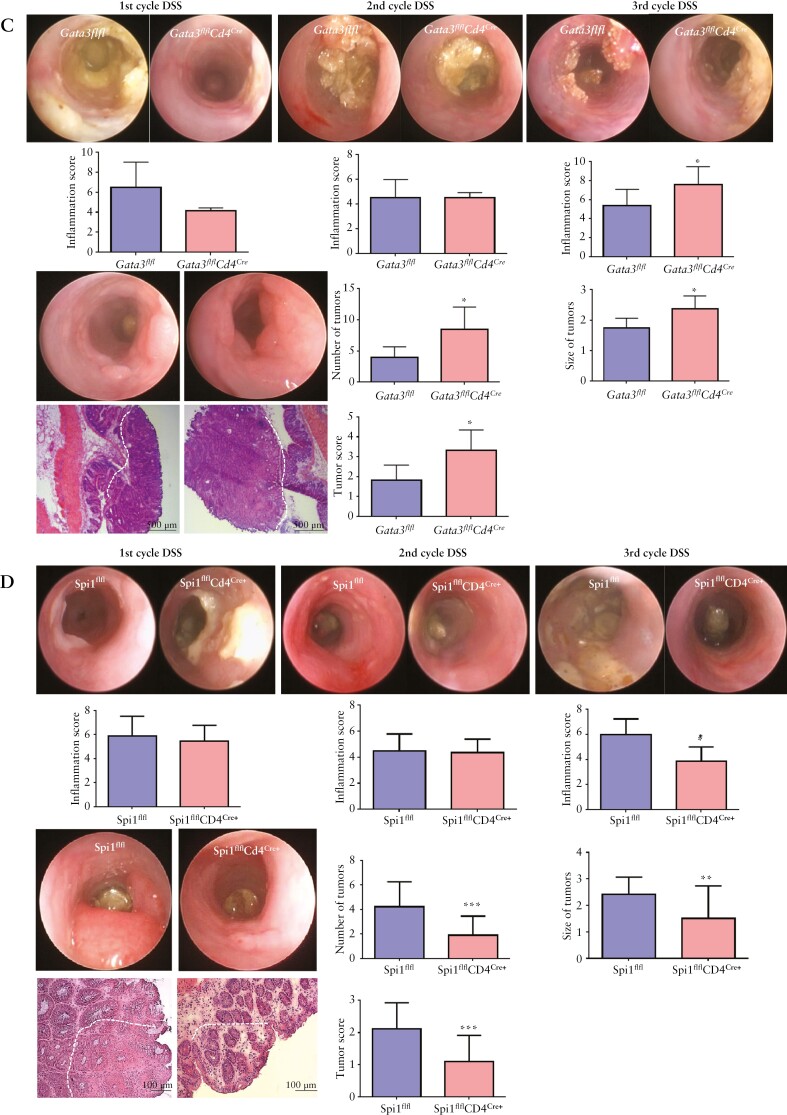
Function of GATA3^+^ T cells and PU.1^+^ T cells in experimental CRC. A] CD4^+^ LPMCs from tumour and control tissue of AOM/DSS-treated wild-type mice were analysed by FACS for the expression of GATA3 and PU.1. Representative scatter plots are shown. Significant differences are indicated [*p <0.05; ***p <0.001]. Results combine values from four mice per group. Gating strategy is shown in Supplementary Figure 1. B] Cryosections of colitis-associated colonic tumours from wild-type and IL-9 KO mice stained with an anti-PU.1 antibody [green], an anti-CD4 antibody [red], and DAPI. Statistical analysis of three mice per group is shown [**p <0.01; ***p <0.001]. C] Conditional GATA3 KO mice were analysed in the AOM/DSS model. Analysis of inflammation after each DSS cycle, tumour number, tumour size and tumour score were determined. Shown are representative pictures of seven mice per group and significant differences [*p <0.05]. The tumour margin is highlighted by a dashed line. D] Miniendoscopic analysis of mice with conditional inactivation of PU.1 in T cells treated with AOM/DSS. Inflammation scoring, tumour growth and number analysis were performed. Data represent results of three independent experiments with five mice per group. Significant differences are indicated [*p <0.05; **p <0.01; ***p <0.001]. The tumour margin is highlighted by a dashed line. CRC, colorectal cancer; AOM, azoxymethane; DSS, dextran sodium sulphate; LPMCs, lamina propria mononuclear cells.

We further analysed the transcriptional mechanisms driving IL-9-dependent tumorigenesis. As Th9 cells can be induced by PU.1- and GATA3-dependent IL-9 gene transcription,^22^ we studied the expression of these transcription factors in lamina propria cells from AOM/DSS-treated wild-type mice. We found more PU.1^+^ T cells in the tumour tissue than GATA3^+^ cells [Figure 4A]. Compared with control tissue, the number of PU.1^+^ T cells was significantly increased [Figure 4A, B], underlining their crucial role in colitis-associated tumours.

To analyse the influence of GATA3 in colitis-associated tumour development, we used conditional GATA3-floxed mice that were crossed to *Cd4*^cre^ mice. These mice, with a T cell-specific GATA3 deletion, were equally inflamed compared with littermate controls during the first two DSS cycles, but showed augmented colitis activity during the third cycle of DSS. We found a higher tumuor burden when GATA3 was absent in T cells [Figure 4C]. Next, we studied the effects of PU.1 deficiency and created T cell-specific PU.1-deficient mice by crossing *Cd4*^cre^ mice with floxed *Spi1* [encoding PU.1] animals. Similarly to IL-9 inactivation, deficiency of PU.1 in T cells suppressed DSS colitis after the third cycle of DSS treatment. Additionally, we detected a significantly decreased tumour burden in *Spi1*^*flfl*^*Cd4*^cre^ mice [Figure 4D], which indicates once more a protumorigenic role of Th9 cells in colorectal neoplasias.

### 3.4. IL-9 regulates production of the proinflammatory cytokine IL-6 in mucosal Th9 lymphocytes

**Figure 5.**
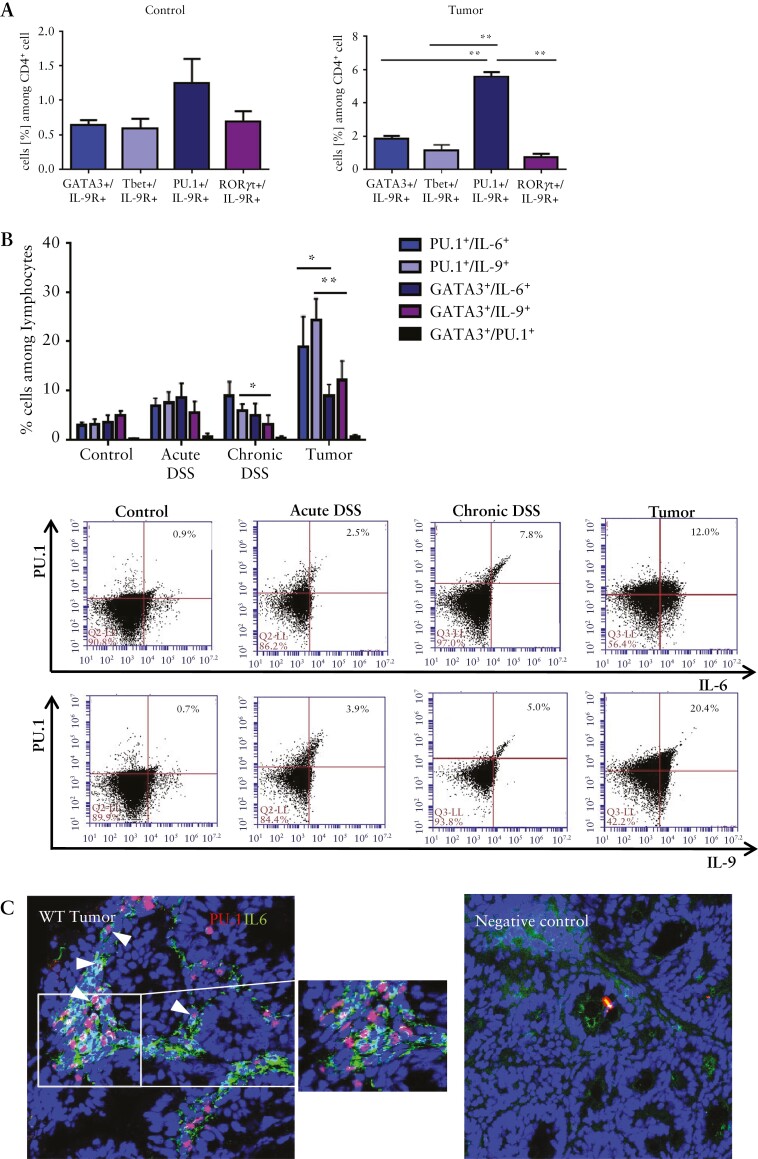

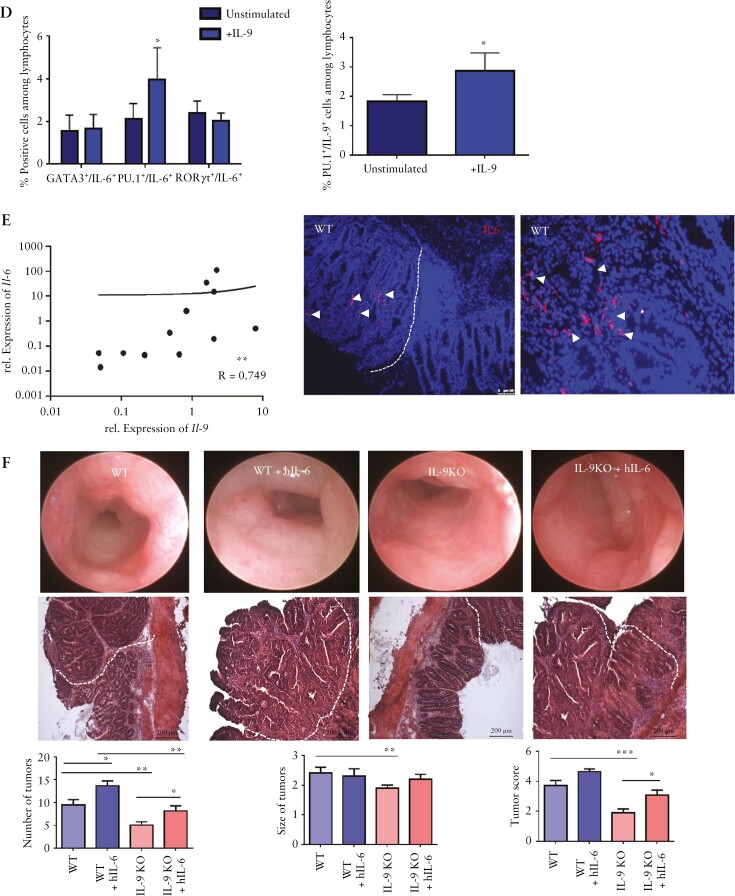
PU.1 controls IL-6 production by T cells. A, Isolated LPMCs from tumour tissue of AOM/DSS-treated wild-type mice and control tissue were analysed by FACS for the IL-9 receptor expression on GATA3^+^, Tbet^+^, PU.1^+^, and RORγt^+^ CD4^+^ T cells. Statistical analysis of two independent experiments is shown with significant differences [**p <0.01]. B] FACS analysis of LPMCs isolated from AOM/DSS-treated wild-type mice and IL-9 knockout mice was performed. Cells were isolated after first and third cycle of DSS treatment and after tumour development. Six mice per group were analysed for the expression levels of PU.1/IL6, PU.1/IL9, GATA3/IL-6, GATA3/IL-9, and PU.1/GATA3 in CD4^+^ T cells. Representative FACS images are shown. Gating strategy is represented in Supplementary Figure 1. C] Cryosections of tumour tissue from AOM/DSS-treated wild-type mice [*n* = 3] were analysed by double staining for PU.1 [red] and IL-6 [green]. Representative stainings are shown. Double-positive cells are highlighted by arrowheads. D] Isolated LPMCs from wild-type mice were stimulated for 2 days with anti-CD3/CD28 antibodies and 1 µg IL-9 before analysis by FACS. Significant differences from three independent experiments are indicated [*p <0.05]. E] Total mRNA from tumour tissue of AOM/DSS-treated wild-type mice was analysed for the expression of IL-6 and IL-9. The correlation coefficient between IL-6 and IL-9 levels is indicated [left panel]. Immunofluorescence staining for IL-6 was done on cryosections from wild-type and IL-9 KO mice. Representative stainings from three mice per group are shown. F] AOM/DSS-treated wild-type and IL-9 KO mice were additionally injected with hyperIL-6 [hIL-6]. H&E stainings and statistical analysis of two independent experiments are shown [*p <0.05, **p <0.01; ***p <0.001]. CRC, colorectal cancer; AOM, azoxymethane; DSS, dextran sodium sulphate; LPMCs, lamina propria mononuclear cells; H&E, haematoxylin and eosin; KO, knockout.

To investigate specific target cells for the pro-inflammatory cytokine IL-9, we checked for the expression of the IL-9 receptor. We isolated LPMCs from control tissue and tumour tissue of wild-type mice and analysed different T helper cell subsets. We found that distinct T helper subtypes express the IL-9 receptor. In contrast to T-bet, GATA3- and RORγt-expressing T cells, PU.1-expressing Th9 cells in tumour tissue expressed significantly more IL-9 receptor [Figure 5A]. Additionally, we saw that mucosal Th9 cells not only produce IL-9 but also the pro-inflammatory cytokine IL-6 [Figure 5B]. IL-6-expressing T cells increased during the different stages of tumour development from control tissue to tumour tissue. We observed significantly more PU.1-expressing T cells producing IL-6 and IL-9 than GATA3-expressing T cells [Figure 5B]; this shows that PU.1^+ ^Th9 cells are a key source of IL-6 production in colorectal neoplasias. Double staining for PU.1 and IL-6 confirmed that many PU.1^+^/IL-6^+^ cells are present in the mucosa of the tumour-bearing wild-type mice [Figure 5C]. Finally, we searched out that IL-9 stimulation of PU.1^+ ^Th9 cell caused increased production of IL-6 and an increased percentage of IL-9-producing cells [Figure 5D]. These findings suggested the presence of an auto-regulatory loop in which mucosal Th9 cells produce IL-9, express IL-9R, and generate increased levels of cytokines such as IL-6 upon IL-9 exposure.

As IL-6 is a protumorigenic cytokine that is highly elevated in colitis-associated neoplasias,^12,15^ we checked the correlation between *Il-6* and *Il-9* mRNA levels in colorectal tumours of wild-type mice. *Il-6* and *Il-9* expression positively correlated in AOM/DSS tumour tissue. Furthermore, high numbers of IL-6^+ ^cells were evident in the lamina propria of the tumour tissue from wild-type mice [Figure 5E]. These findings confirmed that IL-6 levels are induced in AOM/DSS tumour tissue and suggested the possibility that reduced IL-6 levels may contribute to the protective effects of IL-9 deficiency. Therefore, we focused on the influence of IL-6 on tumour development in IL-9-deficient animals. We treated IL-9 knockout mice during AOM/DSS procedures with hyperIL-6, a designer fusion protein consisting of the soluble IL-6 receptor and IL-6.^34^ Strikingly, IL-9-deficient mice given hyperIL-6 showed an induction and development of colorectal tumours comparable to wild-type mice [Figure 5F]. Thus, the induction of IL-6 function reversed the protective phenotype of IL-9 knockout mice and restored tumour induction to levels indistinguishable from wild-type mice.

### 3.5. IL-9 regulates IL-9 receptor expression and, together with IL-6, controls proliferation of intestinal epithelial cells in colorectal neoplasias

**Figure 6.**
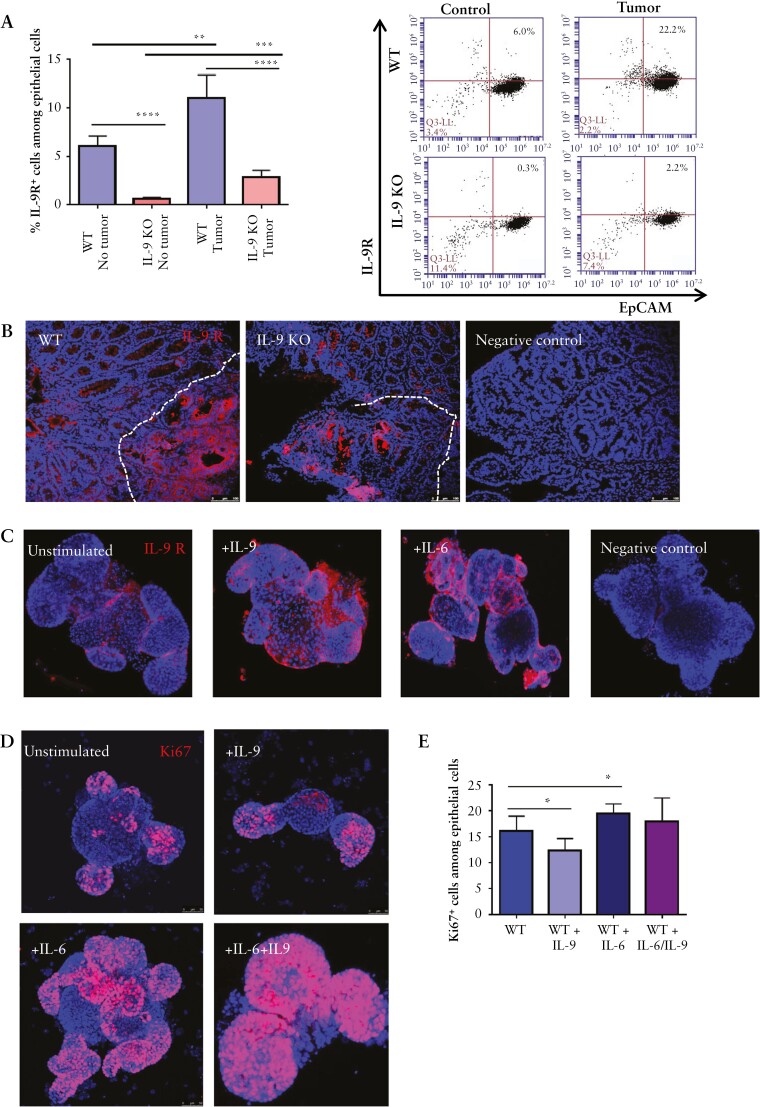
IL-6 and IL-9 effects on epithelial cells during tumorigenesis. A] Isolated epithelial cells from AOM/DSS treated wild-type and IL-9-deficient mice were analysed for the expression of the IL-9 receptor. Significant differences [five mice per group] are indicated [**p <0.01; ***p <0.001] and gating scheme is shown in Supplementary Figure 1. B] Cryosections of tumour tissue from wild-type and IL-9 KO mice were stained for the IL-9 receptor [red]. Dotted lines indicate the tumour region. C] Organoid cell cultures from wild-type animals were stimulated with 100 ng rmIL-9, 50 ng hIL6, or left untreated. Crypts were stained for the IL-9 receptor and cell nuclei with DAPI. Representative stainings from one crypt out of three experiments are shown. D] Immunofluorescence staining of the proliferation marker Ki67 in organoids from wild-type mice. Organoids were stimulated with IL-6 and IL-9, as indicated. Six organoids were stained per group. E] Organoids were stimulated with IL-6, IL-9, or both cytokines for 2 days, subsequently stained for Ki67, and analysed by FACS. Statistical analysis [*n* = 6] is shown and significant differences are indicated [*p <0.05]. AOM, azoxymethane; DSS, dextran sodium sulphate; KO, knockout.

IL-9 signalling has been previously shown to regulate the activation of intestinal epithelial cells [IEC] under homeostatic conditions.^24,25^ We therefore explored the expression of the IL-9 receptor on IECs in tumour tissue. Flow cytometry analysis clearly showed an upregulation of IL-9R expression in the tumour tissue of wild-type and IL-9 knockout mice as compared with control tissue lacking tumours [Figure 6A, B]. However, expression levels were markedly lower in IL-9 knockout mice. Immunofluorescence staining for IL-9R confirmed expression on epithelial cells of tumour tissue in wild-type mice [Figure 6B]. As these findings suggested the possibility that IL-9 induces IL-9R expression in intestinal epithelial cells, we next determined the functional effects of IL-9 signalling on IL-9R expression by *in vitro* studies using tumour organoids. Staining by immunofluorescence for IL-9R revealed that IL-9 and IL-6 are able to upregulate IL-9R expression on epithelial cells [Figure 6C], suggesting that both cytokines may augment functional IL-9 effects on IECs.

For further functional analysis of IL-9 signalling on IECs, we next focused on cell proliferation. We noticed that IL-9 stimulation downregulated IEC proliferation, whereas IL-6 upregulated proliferation of epithelial cells [Figure 6D]. The combination of both pro-inflammatory cytokines induced a higher Ki67 expression in organoids and abrogated the negative effect of IL-9 on proliferation of epithelial cells [Figure 6D, E].

### 3.6. IL-9 affects STAT3/SOCS3 signalling in epithelial cells and impairs barrier function

**Figure 7.**
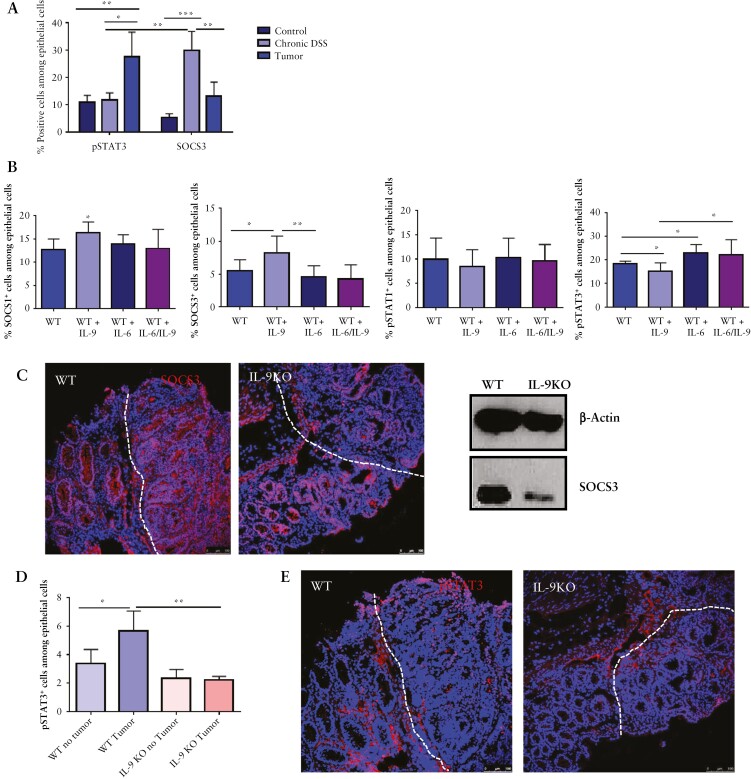

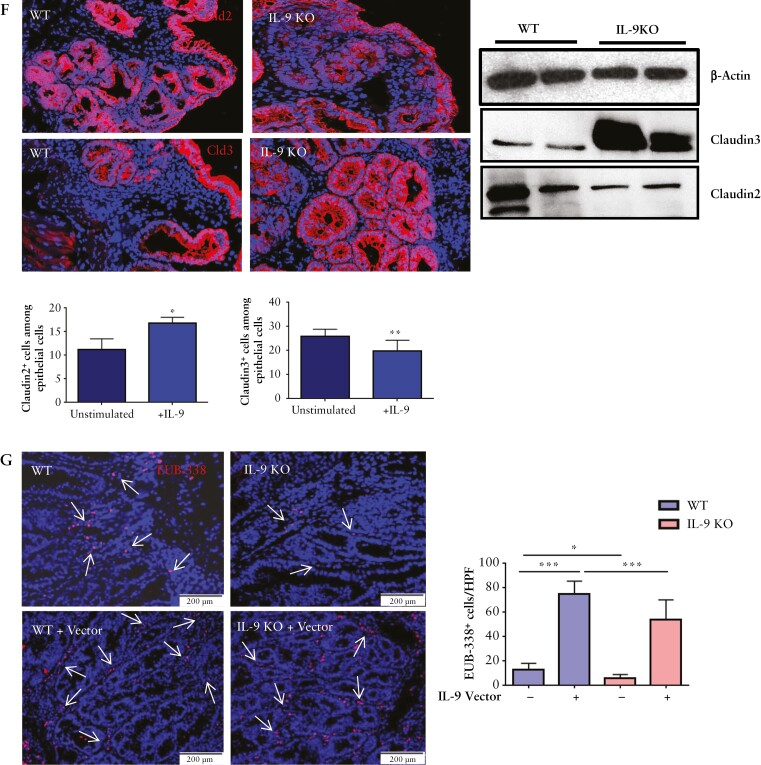
IL-9 effects on epithelial cells of colitis-associated neoplasias. A] Assessment of SOCS3 and pSTAT3 expression during CAC. Isolated epithelial cells from WT mice were investigated by FACS during chronic DSS inflammation and after tumour formation and compared with healthy controls. Results from four mice per condition and significant differences are indicated [*p <0.05; **p <0.01; ***p <0.001]. B] FACS analysis of organoids incubated with either 100 ng IL-9, 100 ng IL-6, or the combination of both cytokines. Organoids were stained for the epithelial marker EpCAM and the intracellular markers SOCS1, SOCS3, pSTAT1, or pSTAT3. Statistical analysis of six different experiment is shown [*p <0.05; **p <0.01]. C] Left panels: cryosections from AOM/DSS-treated wild-type and IL-9 knockout mice were stained for the expression of SOCS3. Nuclei were stained with DAPI. Representative stainings of three mice per group are shown. Right panel: isolated epithelial cells from tumour tissue of wild-type and IL-9 KO mice were analysed by western blot for SOCS3 expression with β-Actin as loading control. D] Isolated epithelial cells of tumour tissue and normal tissue from wild-type and IL-9-deficient mice were analysed by FACS for the expression of pSTAT3. Statistical analysis with significant differences [*p <0.05; **p <0.01] is shown [four mice per group]. E] Cryosections from wild-type and IL-9 knockout mice were stained with an anti-pSTAT3 antibody and cell nuclei with DAPI. pSTAT3^+ ^cells were detected mostly in the tumour tissue of wild-type mice. Representative stainings are shown [three mice per group]. F] Expression of claudin tight junctions was analysed on cryosections of tumour tissue from wild-type and IL-9 knockout mice. Representative pictures are shown for claudin-2 [Cld2] and claudin-3 [Cld3] [left panels]. Epithelial cells from tumour tissue of wild-type and IL-9 deficient mice were analysed by western blots for the protein expression of claudins with β-Actin as loading control [right panel]. G] FISH analysis for the assessment of bacterial translocation in wild-type and IL-9 knockout mice. Cryosections were stained with the EUB-338 probe to detect bacteria [arrows]. Representative stainings are shown from one mouse out of four mice per group. Statistical analysis of EUB-338^+ ^cells per HPF is shown [*p <0.05; ***p <0.001]. CAC, colitis-associated cancer; AOM, azoxymethane; DSS, dextran sodium sulphate; WT, wild-type; HPF, high-power field.

We focused on potential IL-9 target genes in the epithelium with regard to cell proliferation. In the AOM/DSS model, we found an induction of pSTAT3 levels in tumour tissue of wild-type mice as compared with colitic and control mice [Figure 7A]. The STAT3 inhibitor SOCS3 was significantly suppressed during tumour development as compared with tissue with mucosal inflammation lacking tumours. The pSTAT3/SOCS3 axis is markedly altered during colorectal tumorigenesis and might be a target for IL-9-dependent regulation of epithelial cell proliferation. To further investigate potential mechanistic effects, we incubated wild-type organoids either with IL-9, IL-6, or a combination of both cytokines. IL-9 alone upregulated SOCS1 and SOCS3 expression in epithelial cells and reduced pSTAT3 levels [Figure 7B]. In contrast, IL-6 upregulated pSTAT3 rather than SOCS1 and SOCS3 expression levels. The combination of IL-9 and IL-6 abrogated the induction of SOCS1 and SOCS3 levels by IL-9 and induced pSTAT3. However, the signalling pathway through STAT1 was not affected by IL-6 or IL-9 [Figure 7B]. These results demonstrated that IL-6 and IL-9 are important regulators of the pSTAT3/SOCS3 signalling pathway and may control IEC proliferation during tumorigenesis. Consistently, IL-9 deficiency was associated with reduced levels of SOCS3 and pSTAT3 [Figure 7C–E], probably due to the suppression of the IL-9/IL-6 axis driving IEC proliferation.

To determine potential effects of IL-9 on barrier function, we analysed the expression of tight junction proteins. IL-9 deficiency in colorectal neoplasias led to lower levels of the pore-forming tight junction protein claudin-2 [Figure 7F]. The sealing protein claudin-3, however, was induced in IL-9 deficient animals, thus underlining that the absence of IL-9 strengthens the barrier function *in vivo*. Conversely, IL-9 treatment induced claudin-2 but reduced claudin-3 levels in IECs. Finally, we assessed barrier function in wild-type and IL-9-deficient mice in colorectal neoplasias by measuring bacterial translocation. We found a significant reduction of bacteria in AOM/DSS-treated IL-9-deficient animals [Figure 7G]. IL-9 overexpression led to a significant induction of bacterial translocation; this shows that IL-9 controls the barrier function in colitis-associated neoplasias *in vivo*. Thus, IL-9 controls tumour cell proliferation in colorectal neoplasias via IL-6 and impairs barrier function markedly.

## 4. Discussion

Previous studies highlighted a specific role of Th9 cells in controlling anti-tumour immune responses in solid tumours such as melanomas.^26,27^ In contrast, we uncovered in the present study a key protumoral role of Th9 cells and IL-9 in colorectal cancer [CRC] and colitis-associated neoplasias. CRC patients showed elevated IL-9 and PU.1 levels in mucosal T cells, indicating an enrichment of Th9 cells in the tumour tissue. Furthermore, we analysed colitis-associated tumours and detected even higher levels of Th9-associated factors and marked correlations between *GATA3*, *SPI.1*, and *IL-9* mRNA levels, suggesting the relevance of Th9 cells in these patients. Functional studies in the murine DSS and AOM/DSS models identified an important role of IL-9 and Th9 cells in driving colitis activity and tumour growth *in vivo*. Mechanistic experiments demonstrated that IL-9 signalling controls IL-6 production by tumour-infiltrating T cells, leading to IL-6-dependent pSTAT3 activation and proliferation of intestinal epithelial cells [IEC]. Moreover, IL-6 production may enhance mucosal inflammation via T cell activation,^6,35,36^ providing a link between inflammation and tumour growth. In agreement with this link, chronic inflammation and flares of disease have been identified as risk factors for CAC.^37–39^ Our findings suggest that Th9 cells regulate pro-inflammatory and pro-tumoral signalling pathways between tumour-infiltrating T cells and IEC, and therefore these cells emerge as a key regulator of colorectal tumorigenesis.

The major hallmarks of the microenvironment control the tumour progression in CRC.^3,10,40,41^. Densities of CD3^+ ^and cytotoxic CD8^+ ^T cells in the tumour and the invasive margins are key prognostic markers in CRC.^42^ Moreover, cytokines produced by T cells affect CRC growth and prognosis. Alterations in Th1 and Th2 cytokine production regulate the frequency of tumorigenesis in the colon. For instance, the absence of IFN-γ led to an augmented tumour growth in the AOM/DSS model, thereby affecting anti-tumour immune responses.^43^ Conversely, Th1 signatures were associated with an improved prognosis in CRC patients.^44^ However, Th17 clusters predicted a poor prognosis.^44^ IL-23 and IL-17 favoured barrier defects and tumour growth in experimental colorectal cancer models.^45^ In contrast to these findings on Th1 and Th17 cytokines, little is known about the role of Th9 cells in CRC. Here, we showed that PU.1-expressing Th9 cells are induced in patients with CRC and play a dominant protumoral role in the experimental CRC model. In contrast, GATA3^+ ^T cells had no effect on tumour growth *in vivo*. Moreover, the transcription factor PU.1, rather than IRF4, GATA3, or STAT6, is the main driver for Th9 cell differentiation.^20, 21^ Thus, PU.1-driven Th9 cells, rather than GATA3-expressing T cells, are essential for tumorigenesis. Previous studies demonstrated marked pro-inflammatory effects of IL-9 and protective effects of IL-9 deficiency in acute and chronic oxazolone-induced colitis.^24^

In contrast, we noted here that IL-9 deficiency had a mild anti-inflammatory effect in chronic DSS colitis only after the third DSS cycle, suggesting that IL-9 has a limited pro-inflammatory capacity in the AOM/DSS model under our experimental conditions. The reasons for these differences are not entirely clear but might be related to differences between the colitis models, the microbial factors driving colitis activity, or different cytokine patterns. However, this observation suggested that IL-9 deficiency has limited effects on tumour growth by regulating the mucosal inflammation but may directly modulate tumour growth via a decreased pro-inflammatory cytokine millieu. Indeed, mechanistic studies revealed that Th9 cells express IL-9R and respond to IL-9 stimulation with the production of the pro-tumorigenic cytokine IL-6. IL-6 has been previously identified as a key cytokine that directly induces tumuor growth in CRC via STAT3 activation in IECs.^12,15,46,47^. As tumour cells in experimental CRC lose IL-6R expression and become susceptible to IL-6 trans-signalling via the soluble IL-6R,^15^ our findings underlined that Th9 cells may regulate tumour growth via IL-6 trans-signalling. Indeed, treatment of IL-9 knockout mice with hyperIL-6 abrogated the protective effects of IL-9 deficiency and led to a marked tumour growth, thereby demonstrating that IL-9 is an important regulator of IL-6 trans-signalling effects in colorectal neoplasias.

The *IL-6/JAK/STAT3* signalling pathway plays an important role in human malignancies such as CRC.^12,15,46,48,49^ One of the downstream target genes of this signalling cascade is the negative feedback regulator suppressor of cytokine signalling 3 [*SOCS3*] that inhibits STAT3 activation in CRC.^50,51^ Therefore, we studied the effects of IL-6 and IL-9 on the *STAT3/SOCS3* pathway in IECs. Whereas IL-9 was a potent inducer of SOCS3, IL-6 stimulation resulted in marked pSTAT3 activation. In the presence of both IL-9 and IL-6, however, IL-9-dependent effects on SOCS3 were suppressed and STAT3 was activated. Thus, the pro-inflammatory cytokines IL-6 and IL-9 together were able to stimulate proliferation of IECs, thus explaining the pro-tumoral role of these cytokines in CRC. Additional studies in IL-9 knockout mice revealed that IL-9 controls the balance between the sealing protein claudin-3 and the pore-forming claudin-2 protein, indicating that IL-9 impairs barrier function. Whereas claudin-3 has been shown to control malignancy and metastasis in CRC,^52,53^ claudin-2 led to a leaky epithelium in tumour tissue.^54^ Consistent with this concept, IL-9-induced barrier changes led to translocation of commensal bacteria, as shown by FISH studies. This impairment of barrier function during tumorigenesis may contribute to IL-9 effects in tumour development, as gut bacteria favour protumoral mucosal Th17 signatures and local inflammation in colorectal neoplasias.^45^ Mucosal inflammation in turn may further induce the expansion of microorganisms with genotoxic capabilities which favour tumour progression in CRC.^4^

**Figure 8.**
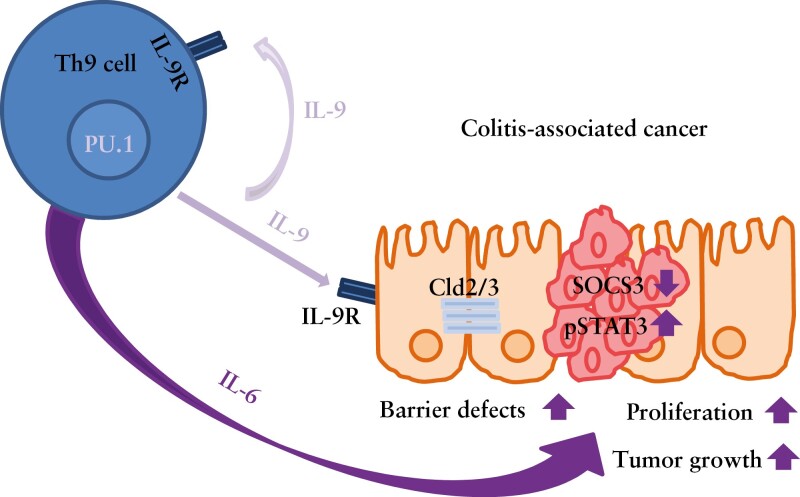
IL-9 function in CRC. Schematic model of IL-9-producing Th9 cells during chronic inflammation leading to an enhanced tumour growth, increasing proliferation of epithelial cells and inducing barrier defects of the epithelial layer.

Our experimental results uncover a novel role of IL-9 and Th9 cells in driving tumorigenesis in CRC. This observation is in contrast to previous findings demonstrating a role of Th9 cells in controlling anti-tumour immune responses in solid tumours such as melanomas.^26,27^ The reasons for these differences are not entirely clear but may be related to the local antigen-rich microenvironment of the gut or the presence of local inflammation. Additionally, they could be due to certain differences in the IL-9R expression on tumour cells. Studies of Hodgkin lymphoma identified a pro-tumoral role of IL-9 in IL-9R-expressing target cells.^55^ Similarly, our studies revealed that tumour cells in experimental colorectal neoplasias exhibited a higher IL-9R expression that controls IECs, suggesting that tumuor cells are sensitive to IL-9-mediated signal transduction. Together with IL-6, IL-9 led to tumour cell proliferation [Figure 8], and the targeting of IL-9 by neutralising antibodies suppressed colorectal tumorigenesis. Summarized, our findings underline a crucial role for IL-9 in CRC and identify IL-9 as a highly potential target for therapy.

## Supplementary Material

jjac097_suppl_Supplementary_Figure_1

jjac097_suppl_Supplementary_Figure_21

jjac097_suppl_Supplementary_Figure_22

jjac097_suppl_Supplementary_Figure_23

jjac097_suppl_Supplementary_Figure_3

jjac097_suppl_Supplementary_Figure_41

jjac097_suppl_Supplementary_Figure_42
