# Supplementary figures and images for: PU.1-driven Th9 Cells Promote Colorectal Cancer in Experimental Colitis Models Through Il-6 Effects in Intestinal Epithelial Cells
Source: J Crohns Colitis. 2022 Jul 6;16(12):1893–910. doi: 10.1093/ecco-jcc/jjac097 (PMC10197880; doi:10.1093/ecco-jcc/jjac097)

A

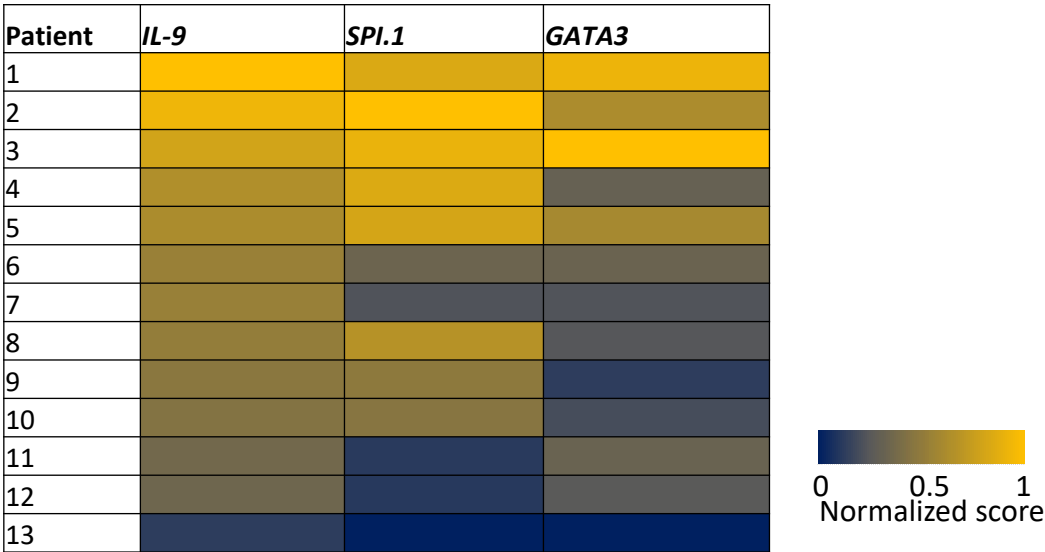

B

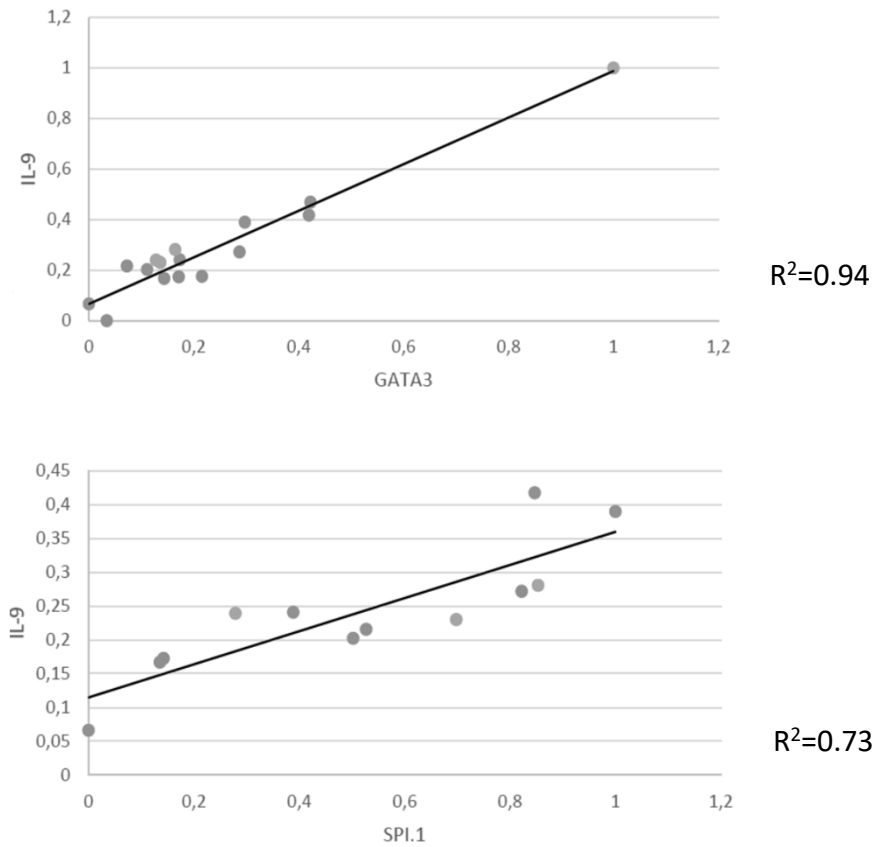

Supplementary Fig. 1

Supplement: jjac097_suppl_Supplementary_Figure_1 [file jjac097_suppl_supplementary_figure_1.pdf]

**A**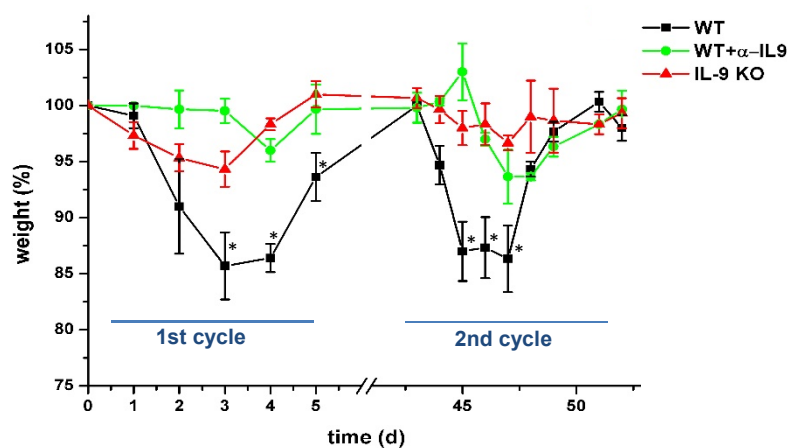**B**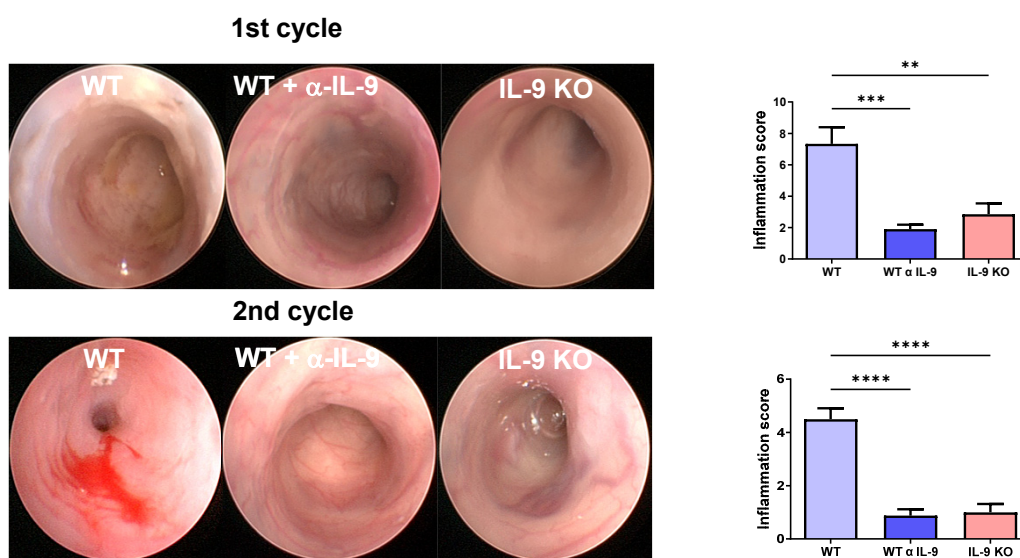**C**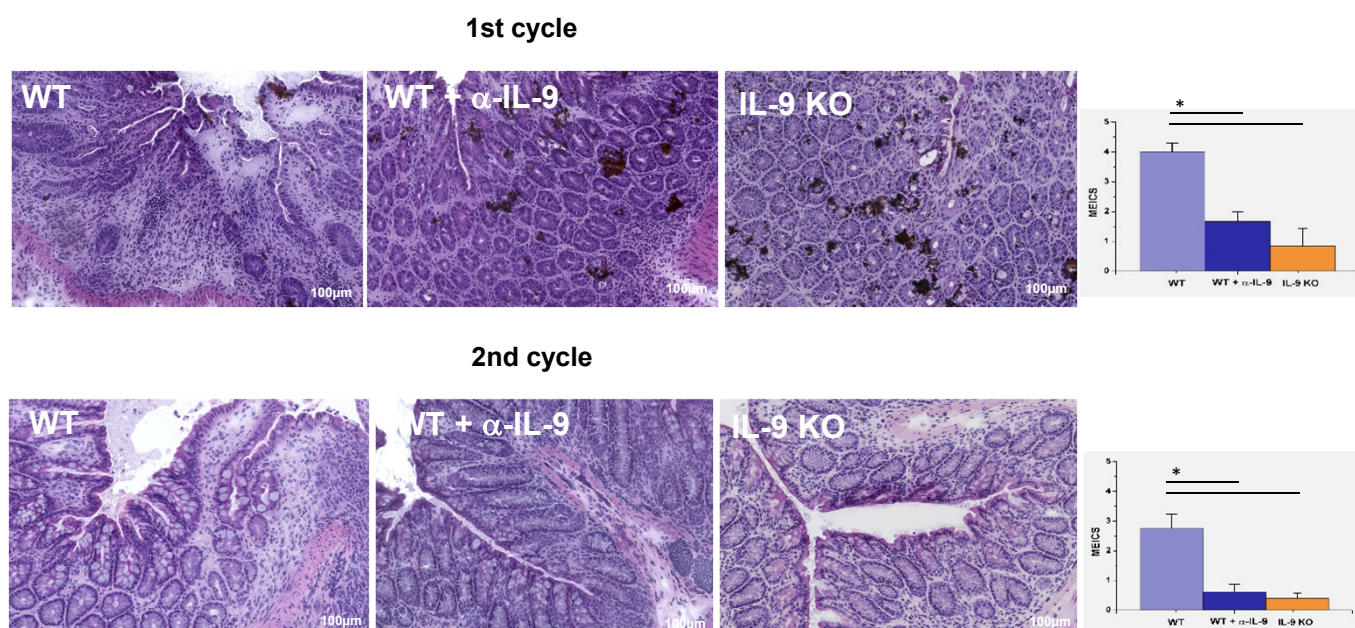**Supplementary Figure 2**

Supplement: jjac097_suppl_Supplementary_Figure_21 [file jjac097_suppl_supplementary_figure_21.pdf]

**D**

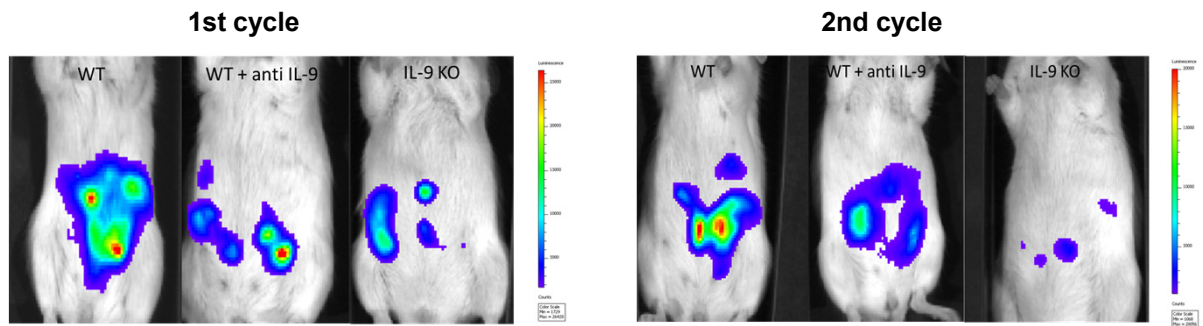

**E**

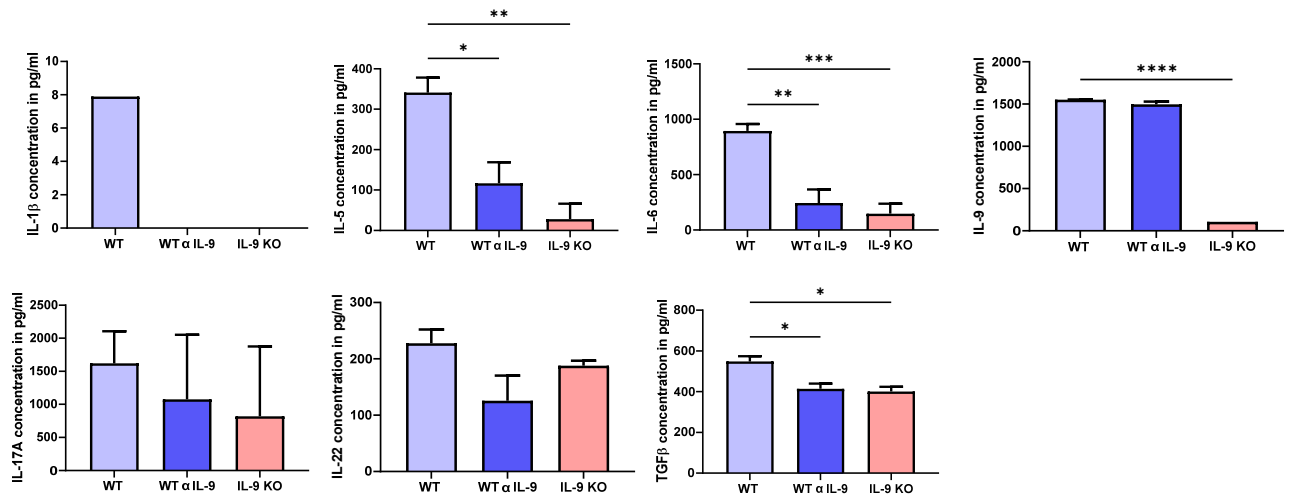

**F**

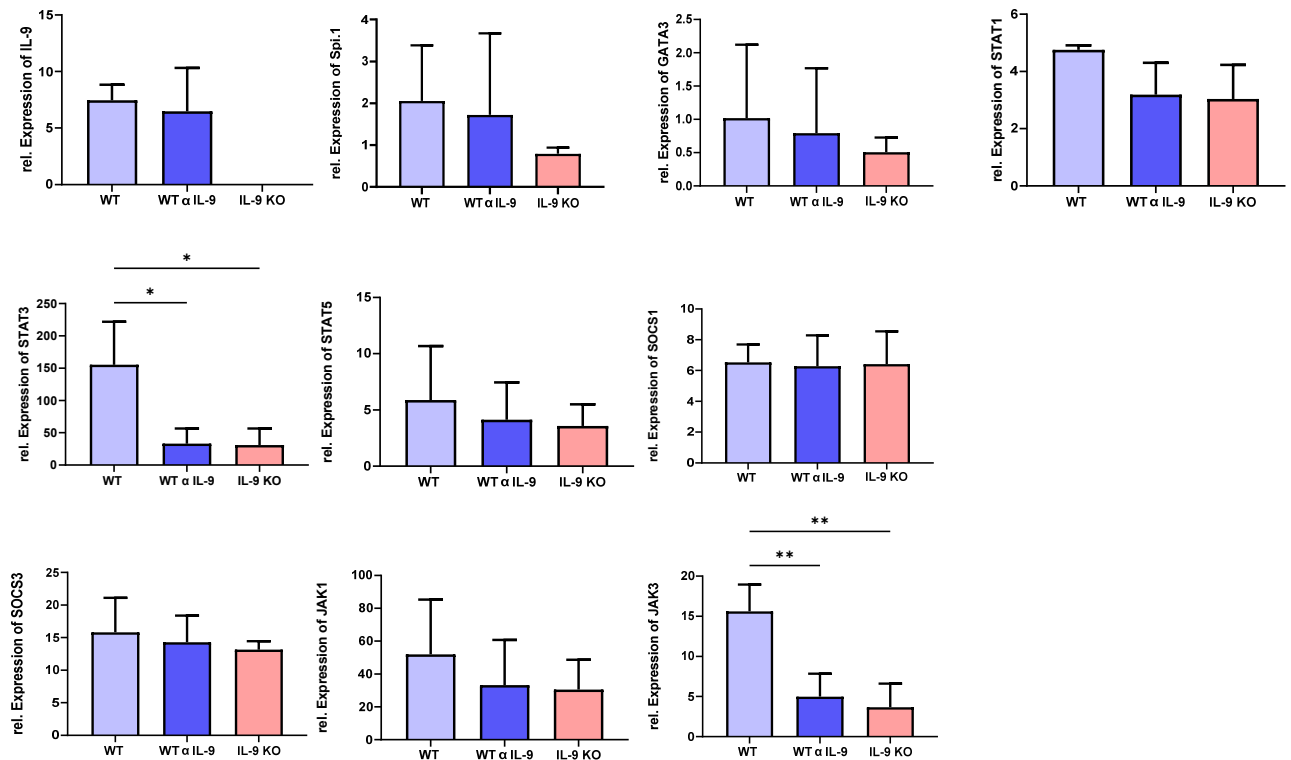

**Supplementary Figure 2**

Supplement: jjac097_suppl_Supplementary_Figure_22 [file jjac097_suppl_supplementary_figure_22.pdf]

**G**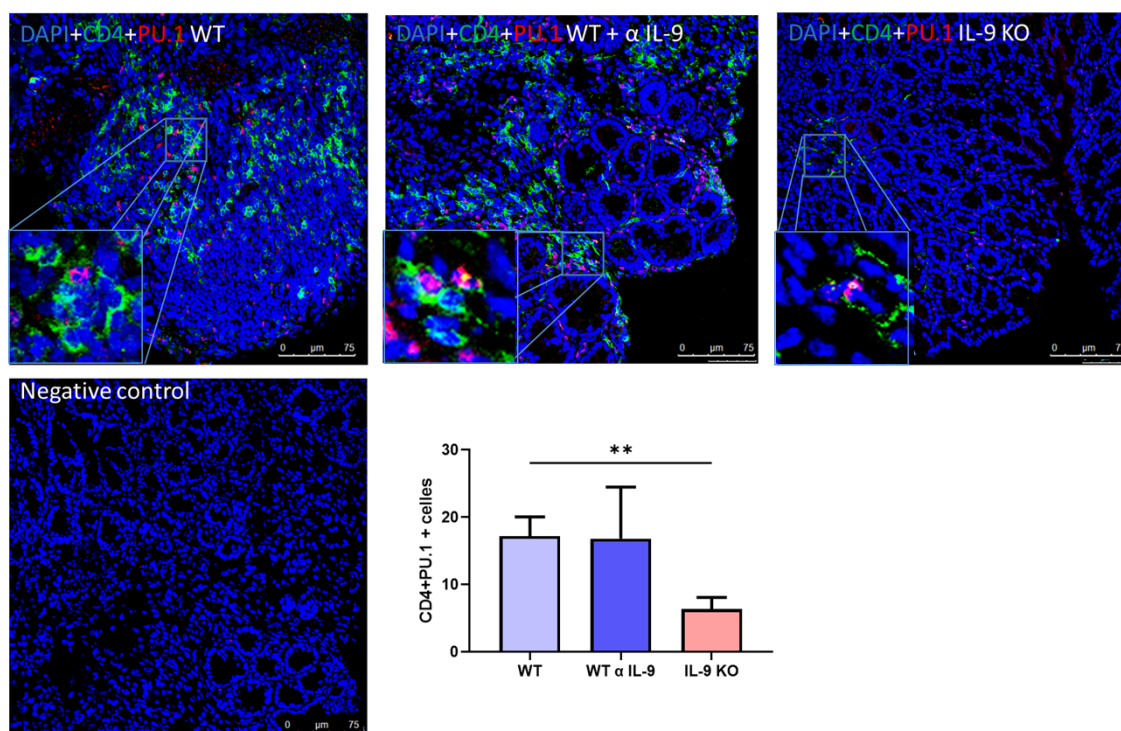

**Supplementary Figure 2**

Supplement: jjac097_suppl_Supplementary_Figure_23 [file jjac097_suppl_supplementary_figure_23.pdf]

Fig. 1B

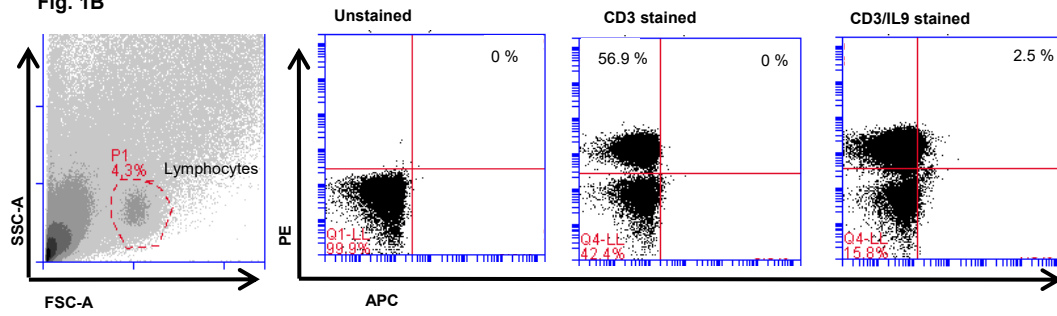

Fig. 4A

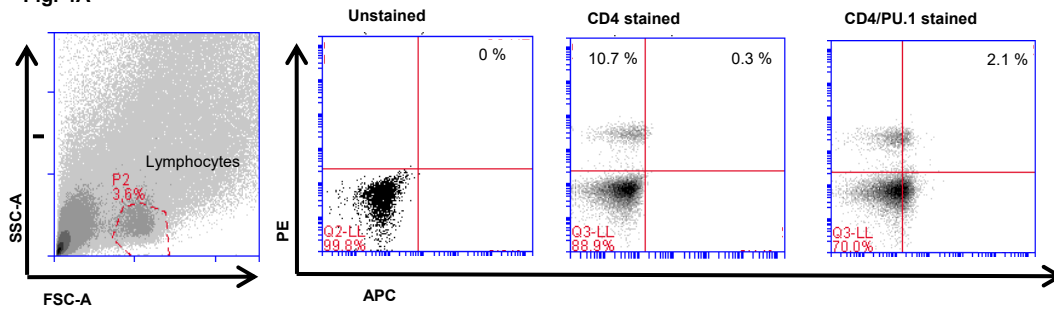

Fig. 5B

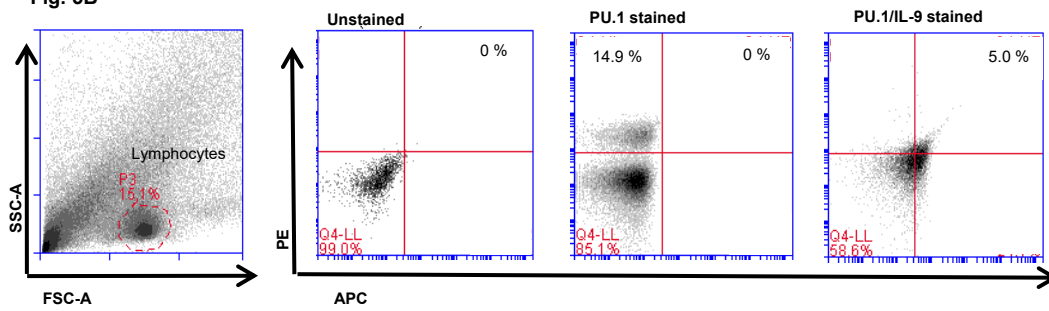

Fig. 6A

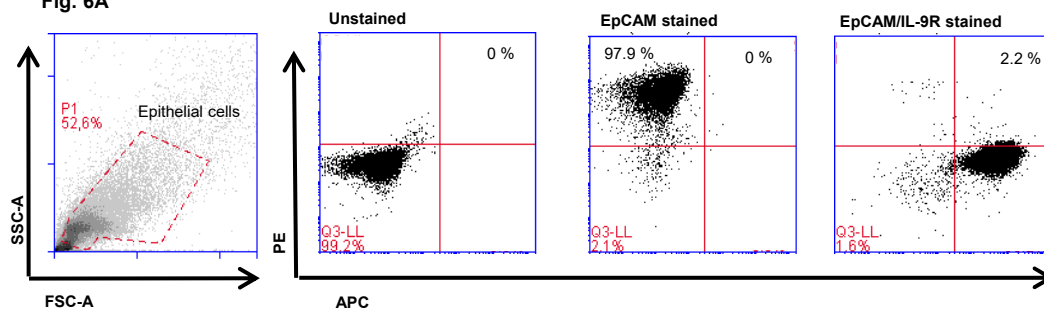

Supplement: jjac097_suppl_Supplementary_Figure_3 [file jjac097_suppl_supplementary_figure_3.pdf]
