# Supplementary material for: PU.1-driven Th9 Cells Promote Colorectal Cancer in Experimental Colitis Models Through Il-6 Effects in Intestinal Epithelial Cells
Source: J Crohns Colitis. 2022 Jul 6;16(12):1893–910. doi: 10.1093/ecco-jcc/jjac097 (PMC10197880; doi:10.1093/ecco-jcc/jjac097)
Supplement: jjac097_suppl_Supplementary_Figure_41 [file jjac097_suppl_supplementary_figure_41.pdf]

antibody	species	application	validation
**Anti CD3**	Human	Immunofluorescence	Ryschich, E., et al., Expression of HLA class I/II antigens and T cell immune response in human neuroendocrine tumors of the pancreas. Tissue Antigens, 2003. 62(1): p. 48-54.
**Anti IL9**	Human	Immunofluorescence	1:50
**Anti CD4**	Human	Immunofluorescence	https://www.biolegend.com/en-us/products/purified-anti-human-cd4-antibody-3650
**Anti PU.1**	Human	Immunofluorescence, FACS	1:200
**Anti CD3**	Mouse	Immunofluorescence	https://www.biolegend.com/en-us/products/purified-anti-mouse-cd3epsilon-antibody-28
**Anti IL-9 R**	Mouse	Immunofluorescence	Gerlach, K., et al., TH9 cells that express the transcription factor PU.1 drive T cell-mediated colitis via IL-9 receptor signaling in intestinal epithelial cells. Nat Immunol, 2014. 15(7): p. 676-86.
**Anti Ki67**	Mouse	Immunofluorescence	https://www.agilent.com/en/product/immunohistochemistry/antibodies-controls/primary-antibodies/ki-67-antigen-(dako-omnis)-76239
**Anti IL-9**	Mouse	Immonufluorescence	http://www.bdbiosciences.com/eu/applications/research/t-cell-immunology/th-9-cells/intracellular-markers/cytokines-and-chemokines/mouse/purified-nale-hamster-anti-mouse-il-9-d9302c12/p/554472
**Anti CD4**	Mouse	Immunofluorescence	Yang, T., et al., Shifts in the Gut Microbiota Composition Due to Depleted Bone Marrow Beta Adrenergic Signaling Are Associated with Suppressed Inflammatory Transcriptional Networks in the Mouse Colon. Front Physiol, 2017. 8: p. 220.
**Anti IL-6**	Mouse	Immunofluorescence	Galie, M., et al., Mammary carcinoma provides highly tumourigenic and invasive reactive stromal cells. Carcinogenesis, 2005. 26(11): p. 1868-78.
**anti-mCD4^{PECy7}**	Mouse	FACS	1:800
**anti-mCD19^{APC}**	Mouse	FACS	1:800
**anti mCD8^{PE}**	Mouse	FACS	1:800
**anti-mEpCAM^{APC}**	Mouse	FACS	Glal, D., et al., ATF3 Sustains IL-22-Induced STAT3 Phosphorylation to Maintain Mucosal Immunity Through Inhibiting Phosphatases. Front Immunol, 2018. 9: p. 2522.
**anti-mEpCAM^{PE}**	Mouse	FACS	1:200
**anti-hCD3^{APC}**	Human	FACS	1:20
**anti-mGATA3^{APC}**	Human/Mouse	FACS	1:20
**anti-mFoxP3^{APC}**	Human/Mouse	FACS	1:200
**anti-mRORgt^{APC}**	Human/Mouse	FACS	Moretti, S., et al., A mast cell-ILC2-Th9 pathway promotes lung inflammation in cystic fibrosis. Nat Commun, 2017. 8: p. 14017.
**anti-mTbet^{APC}**	Human/Mouse	FACS	https://www.labome.com/product/Miltenyi-Biotec/130-098-607.html
**anti-mIL-6^{PE}**	Mouse	FACS	1:800
**anti-mIL-9^{PE}**	Mouse	FACS	1:800
**anti-hIL-9^{PE}**	Human	FACS	https://www.biolegend.com/de-at/products/pe-anti-human-il-9-antibody-1974
**anti-mpSTAT3^{APC}**	Mouse /Human	FACS	1:100
**anti SOCS3**	Mouse	WB	1:1000
**anti Claudin2**	Mouse	WB	0.5-2 µg/mL
**anti Claudin3**	Mouse	WB	Kern, M., et al., Altered Cytokine Expression and Barrier Properties after In Vitro Infection of Porcine Epithelial Cells with Enterotoxigenic Escherichia coli and Probiotic Enterococcus faecium. Mediators Inflamm, 2017. 2017: p. 2748192.

Supplementary Fig. 4
