# Supplementary material for: PU.1-driven Th9 Cells Promote Colorectal Cancer in Experimental Colitis Models Through Il-6 Effects in Intestinal Epithelial Cells
Source: J Crohns Colitis. 2022 Jul 6;16(12):1893–910. doi: 10.1093/ecco-jcc/jjac097 (PMC10197880; doi:10.1093/ecco-jcc/jjac097)
Supplement: jjac097_suppl_Supplementary_Figure_42 [file jjac097_suppl_supplementary_figure_42.pdf]

applicable	Supplier name	Catologue number	clone	Lot number
Hs anti CD3	BioLegend	300402	UCHT1	B242090
Hs anti IL-9	BioLegend	507602	MH9A4	B146870
Hs anti CD4	BioLegend	317402	OKT4	B201306
Hs Mm anti PU.1	invitrogen	MA5-15064	E.388.3	TD2551748
Mm anti CD3	BioLegend	100302	145-2c11	B253421
Mm anti IL-9R	Acris	DM3569P	12F43	180201FP1
Mm anti Ki67	DAKO	M7249	TEC-3	00074047
Mm anti IL-9	BD	554472	D9302C12	6243643
Mm anti CD4	ebioscience	14-0041-85	GK1.5	E03465-1634
Mm anti IL-6	BioLegend	504502	MP5-20F3	B158892
Mm anti Claudin2	invitrogen	516100	polyclonal	1248417A
Mm anti Claudin3	invitrogen	34-1700	polyclonal	1248418A
Mm anti SOCS3	Cell Signaling	2923	polyclonal	2
Anti rabbit Alexa 488	invitrogen	A11008	N/A	1735088
Anti rabbit Alexa 594	invitrogen	A11012	N/A	1704538
Anti rat Alexa 555	invitrogen	A21434	N/A	1783421
Anti rat Alexa 488	invitrogen	A11006	N/A	1423045
rat-anti-mCD4^{PECy7}	BioLegend	100528	RM4-5	B214651
rat-anti-mCD19^{APC}	BioLegend	115511	6D5	B160517
rat-anti mCD8^{PE}	BioLegend	100708	53-6.7	B234269
rat-anti-mEpCAM^{APC}	ebioscience	17-5791-82	G8.8	B217174
rat-anti-mEpCAM^{PE}	BioLegend	118206	G8.8	B214526
mouse-anti-hCD3^{APC}	BioLegend	317318	Okt 03	B263368
rat-anti-mCD3^{APC}	Biolegend	100312	145-2C11	B237984
rat-anti-mGATA3^{APC}	ebioscience	50-9966-42	TWAJ	2048839
rat-anti-mFoxP3^{APC}	ebioscience	50-5773-82	FJK-16s	B228445
rat-anti-mRORgt^{APC}	Miltenyi	130-103-838	N/A	5180829193
rat-anti-mTbet^{APC}	Miltenyi	130-098-607	N/A	5180829180
rat-anti-mIL-6^{PE}	BD	554401	N/A	7159590
rat-anti-mIL-9^{PE}	BioLegend	514103	RM9A4	B202261
mouse-anti-hIL-9^{PE}	BioLegend	507605	MH9A4	B173059
rat-anti-mpSTAT3^{APC}	ebioscience	17-9033-42	LUVNKLA	4316341
anti-rabbit HRP	Cell Signaling	7074S	polyclonal	26
B-Actin	Santa Cruz	sc-47778	C4	J2915

Supplementary Fig. 4
